# Human isogenic cells of the neurovascular unit exert transcriptomic cell type-specific effects on a blood-brain barrier in vitro model of late-onset Alzheimer disease

**DOI:** 10.1186/s12987-023-00471-y

**Published:** 2023-10-31

**Authors:** Undine Haferkamp, Carla Hartmann, Chaudhry Luqman Abid, Andreas Brachner, Alevtina Höchner, Anna Gerhartl, Bernadette Harwardt, Selin Leckzik, Jennifer Leu, Marco Metzger, Marina Nastainczyk-Wulf, Winfried Neuhaus, Sabrina Oerter, Ole Pless, Dan Rujescu, Matthias Jung, Antje Appelt-Menzel

**Affiliations:** 1 Fraunhofer Institute for Translational Medicine and Pharmacology ITMP, Discovery Research ScreeningPort, 22525 Hamburg, Germany; 2 Institute for Physiological Chemistry, Medical Faculty of the Martin, Luther University Halle-Wittenberg, Hollystrasse 1, 06114 Halle (Saale), Germany; 3 Center Health and Bioresources, Competence Unit Molecular Diagnostics, AIT Austrian Institute of Technology GmbH, Vienna, 1210 Austria; 4 Fraunhofer Institute for Silicate Research ISC, Translational Center Regenerative Therapies (TLC-RT), 97070 Würzburg, Germany; 5 Chair Tissue Engineering and Regenerative Medicine (TERM), University Hospital Würzburg, 97070 Würzburg, Germany; 6 Institute of Legal Medicine, University Hospital, 06112 Halle (Saale), Germany; 7 Department of Medicine, Faculty of Medicine and Dentistry, Danube Private University, Krems, 3500 Austria; 8 Department of Psychiatry and Psychotherapy, Division of General Psychiatry, Medical University of Vienna, Vienna, 1090 Austria

**Keywords:** Apolipoprotein E, Human induced pluripotent cells, Blood-brain barrier, Neurovascular unit, Alzheimer disease, Late-onset Alzheimer disease

## Abstract

**Background:**

The function of the blood-brain barrier (BBB) is impaired in late-onset Alzheimer disease (LOAD), but the associated molecular mechanisms, particularly with respect to the high-risk APOE4/4 genotype, are not well understood. For this purpose, we developed a multicellular isogenic model of the neurovascular unit (NVU) based on human induced pluripotent stem cells.

**Methods:**

The human NVU was modeled in vitro using isogenic co-cultures of astrocytes, brain capillary endothelial-like cells (BCECs), microglia-like cells, neural stem cells (NSCs), and pericytes. Physiological and pathophysiological properties were investigated as well as the influence of each single cell type on the characteristics and function of BCECs. The barriers established by BCECs were analyzed for specific gene transcription using high-throughput quantitative PCR.

**Results:**

Co-cultures were found to tighten the barrier of BCECs and alter its transcriptomic profile under both healthy and disease conditions. In vitro differentiation of brain cell types that constitute the NVU was not affected by the LOAD background. The supportive effect of NSCs on the barrier established by BCECs was diminished under LOAD conditions. Transcriptomes of LOAD BCECs were modulated by different brain cell types. NSCs were found to have the strongest effect on BCEC gene regulation and maintenance of the BBB. Co-cultures showed cell type-specific functional contributions to BBB integrity under healthy and LOAD conditions.

**Conclusions:**

Cell type-dependent transcriptional effects on LOAD BCECs were identified. Our study suggests that different brain cell types of the NVU have unique roles in maintaining barrier integrity that vary under healthy and LOAD conditions.

**Graphical abstract:**

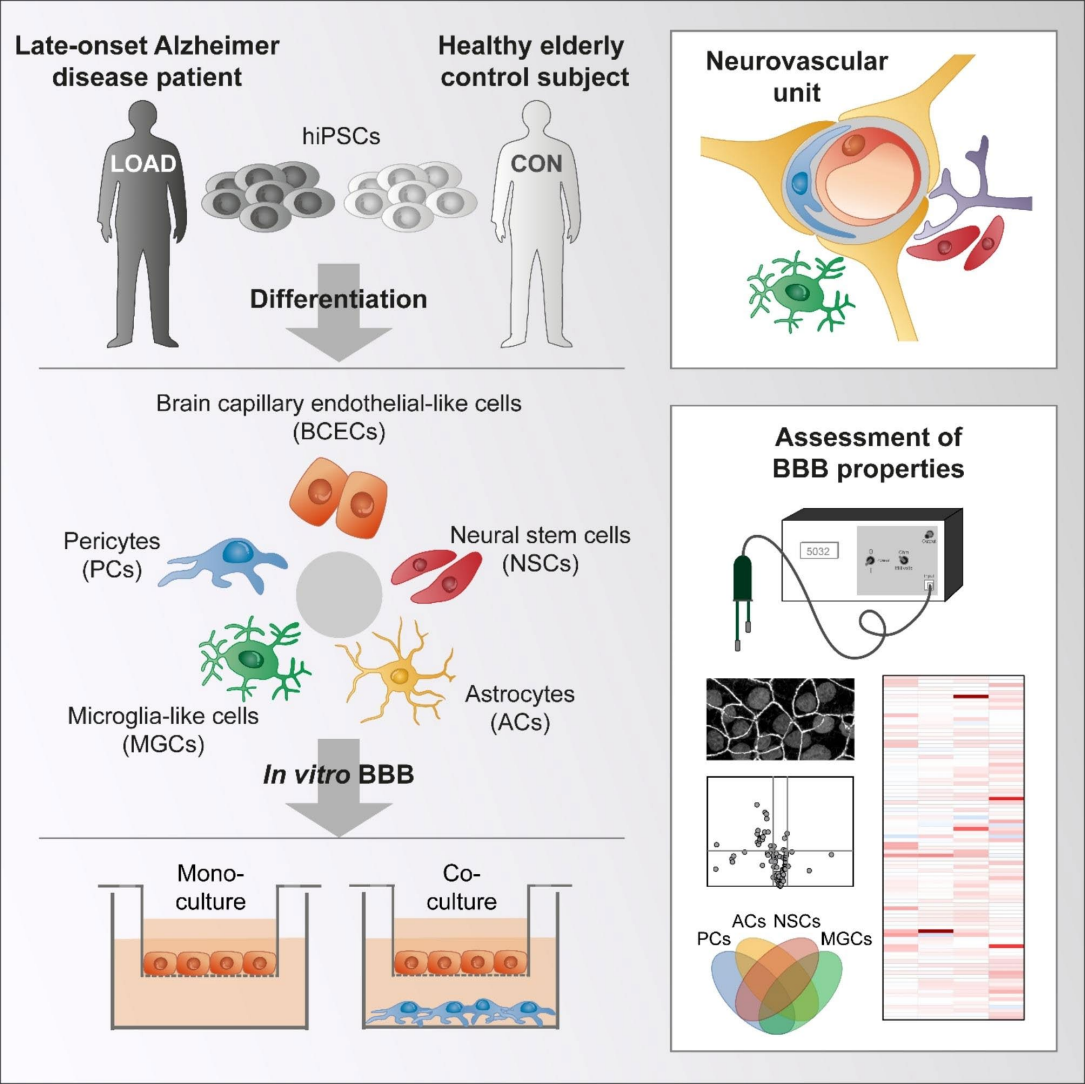

.

**Supplementary Information:**

The online version contains supplementary material available at 10.1186/s12987-023-00471-y.

## Background

According to the World Health Organization, late-onset Alzheimer disease (LOAD) and other forms of dementia were the seventh leading cause of death worldwide in 2019, and the number of affected people is expected to continue to increase in the coming years [1]. The pathophysiology of LOAD is poorly understood, which has contributed to a lack of preventive and curative treatments [2]. LOAD is accompanied by extracellular formation of neuritic plaques consisting of misfolded beta amyloid (Aβ) peptides as well as intracellular accumulation of tau proteins and formation of neurofibrillary tangles. The neurovascular unit (NVU), which consists of specialized brain capillary endothelial cells (BCECs) and other central nervous system (CNS) cells mainly carries out degradation and elimination of these toxic species. Altered deposition of microtubule-associated protein tau (tau pathology) and accumulation of Aβ (cerebral amyloid angiopathy) within or adjacent to BCECs induces a loss of vascular function in LOAD [3–5]. The APOE gene encoding for apolipoprotein E is the most important risk gene for LOAD [6]. The APOE gene contains two single nucleotide polymorphisms that generate three different isoforms (ε2, ε3, and ε4). Carriers of APOE ε4 (APOE4) have a 15-fold increased risk of developing LOAD, while other risk alleles have a much smaller impact. The APOE4 risk allele impairs the functionality of pericytes (PCs) [7, 8] and astrocytes (ACs) [9] and thus also the functionality of the NVU. However, little is known about the impact of the APOE4 isotype on BCECs.

Animal models, in particular rodents, have been used to study the NVU in the context of LOAD but are limited in their ability to model this disease due to species-specific differences in brain anatomy and physiology [10–12]. Accordingly, the understanding of tau pathology is limited, with only Aβ plaques observed as a strong phenotype in mice carrying multiple familial Alzheimer mutations and human APOE [13]. Numerous immortalized cell lines from human and animal origin as well as primary cells have been used to study LOAD in vitro for drug development but there are limitations with respect to their species of origin, the mutated genomes of cell lines, or the limited availability (especially for human CNS cells) and reproducibility of primary cells. Human cell lines cannot adequately mimic cell physiology and may lack important properties of mature tissue cells that are required for the onset, progression, and treatment of LOAD [10–12]. These limitations of existing animal and in vitro models have contributed to the lack of success in developing highly effective drugs that target LOAD. In order to help close the translational gap, we developed a multicellular isogenic model that mimics the NVU in vitro and allows for a comparison between healthy and LOAD conditions based on human induced pluripotent stem cells (hiPSCs).

The blood-brain barrier (BBB) is anatomically a part of the NVU and regulates the transport of harmful or nourishing substances between blood circulation and brain parenchyma [14]. In this process, BCECs have a central role in forming a physical barrier to prevent paracellular transport [15]. Tight junctions (TJs) between the BCECs seal the intercellular space and restrict the unregulated paracellular transport of mainly hydrophilic compounds, and members of the ATP binding cassette (ABC) and solute carrier (SLC) transporter protein families regulate the transcellular transport of small molecules. Thus, the transport and metabolic barrier functions of BCECs contribute significantly to the maintenance of brain homeostasis. However, the maintenance of the barrier is dependent on a complex interplay with supporting PCs, ACs, microglia, and neurons [16]. To better understand this complex crosstalk, different cell types of the NVU have been combined for in vitro modelling of the BBB in health and disease [11]. In addition, the NVU can undergo changes over time which can contribute to the development of age-related diseases including LOAD [17]. The aim of this study was to improve our understanding of the effects induced by individual cell types of the NVU under healthy and LOAD conditions. Based on our previously established BBB model [18], we developed a more advanced multicellular model of the NVU that allows co-culture of BCECs with isogenic ACs, microglia-like cells (MGCs), neural stem cells (NSCs), and PCs by selecting hiPSCs from a LOAD patient and an elderly control subject. For a targeted analysis of BCECs, transcripts that are important for integrity and functionality of BCECs were analyzed using a high-throughput qPCR array that we recently developed [19].

## Methods

### Origin and characterization of hiPSCs

For generation of NVU cells, hiPSCs were derived from a LOAD patient (age 76) who was recruited according to the NINCDS-ADRDA criteria [20] at the outpatient clinic of the Department of Psychiatry, University of Munich, Germany. The patient was diagnosed according to the diagnostic and statistical manual of mental disorders (DSMIV) and carried a homozygous APOE ε4 genotype (APOE4/4). An elderly person (age 64) was selected as a matched control donor. There was an absence of central neurological disease and psychotic disorders in the control subject including first degree relatives, using the Structured Clinical Interview for DSM-IV (SCID I and II) [21] and the Family History Assessment Module (FHAM) [22]. The healthy control individual was matched by ancestry (German), gender, and age and carried a homozygous APOE ε3 genotype (APOE3/3). For the generation of hiPSCs, subjects’ blood was collected and peripheral blood mononuclear cells were isolated for immortalization by Epstein-Barr virus infection. Obtained B-lymphoblastoid cell lines were then used to generate hiPSCs by electroporation with episomal vectors as recently described [23]. The somatic donor cells and resulting hiPSCs were characterized extensively, the latter for their pluripotency characteristics, differentiation capacity, and genomic integrity [23] (https://hpscreg.eu). Recruitment of subjects was approved by the ethics committee of the Hospitals of the Ludwig-Maximilian-University, Munich, which permits anonymous use of material for research purposes, and was carried out in accordance with the Declarations of Helsinki (Project number 275-06).

### Cultivation of hiPSCs

HiPSCs were cultured at 37 °C, 95% humidity, and 5% CO_2_. Cells were cultured in mTeSR^TM^1 (Stemcell Technologies) supplemented with 1% gentamycin (Thermo Fisher Scientific) on Matrigel^TM^-coated 6-well plates (VWR; 0.083 mg/well) in 1 ml Knockout™ DMEM (Thermo Fisher Scientific). For passaging, cells were treated with 1 mg/ml collagenase IV (Thermo Fisher Scientific) in Knockout DMEM for 30 min at 37 °C or Gentle Cell Dissociation Reagent (Stemcell Technologies) for 7 min at room temperature, followed by rinsing once in Knockout DMEM, and subsequently mechanical dissociation and seeding at a 1:10 − 1:100 split ratio with daily medium replacement.

### Isogenic NVU model

HiPSC lines derived from study subjects were used for differentiation into ACs, BCECs, MGCs, NSCs, and PCs. To investigate the influence of co-cultured ACs, MGCs, NSCs, and PCs on BCECs, 5 × 10^4^ cells/cm^2^ were seeded into 24-well plates containing a cell type-specific medium and an appropriate cell type-specific matrix (poly-L-lysin for PCs and ACs; gelatin for MGCs; Matrigel for NSCs). Cells were cultivated in cell type-specific medium for 24 h before these were combined with differentiating BCECs.

BCECs at day 8 of differentiation were sub-cultured using Accutase™ (Thermo Fisher Scientific or Sigma-Aldrich) and seeded at a density of 1 × 10^6^ cells/cm^2^ on top of 1 mg/ml collagen IV (Merck) and 0.5 mg/ml fibronectin-coated (Thermo Fisher Scientific) inserts (24-well plates) and treated for 24 h with 200 µl endothelial cell (EC) medium, consisting of 99.5% human endothelial serum-free medium and 0.25 X (0.5%) B-27™ (all Thermo Fisher Scientific), supplemented with 20 ng/ml hbFGF (Peprotech) and 10 µM retinoic acid (Stemcell Technologies or Sigma-Aldrich) at the apical side. The inserts were transferred into the wells of a 24-well plate containing the appropriate co-culture cell type and with 850 µl neuroglial differentiation (NGD) medium, consisting of Neurobasal™ supplemented with 1X B-27 without vitamin A, 1X N-2 supplement, 2 mM GlutaMAX™ (all Thermo Fisher Scientific), 3.5 ng/ml biotin, 1 mM sodium pyruvate, 0.02% lactic acid solution, 2 mg/ml lipidated bovine serum albumin, 2.5 µg/ml L-ascorbic acid (all from Merck), and 50 mM NaCl (Carl Roth; modified after [24]). The following day (day 10 of BCEC differentiation), apical medium was changed to EC medium without additional growth factors, basolateral NGD medium was refreshed, and the co-culture models were utilized for analytical studies.

### Generation of BCECs

BCECs were generated from hiPSCs as recently described with minor modifications [18, 25–27]. At day -3, hiPSCs were passaged with Accutase and seeded in mTeSR1 supplemented with 10 µM HA100 (Santa Cruz Biotechnologies) or Y-27632 (Stemcell Technologies) on Matrigel-coated 6-well plates using a set number of cells (7.5–12.5 × 10^3^ cells/cm^2^). At day 0, cells were treated with unconditioned medium containing 78.5% DMEM/F12 without glutamine, 20% Knockout serum replacement, 1 mM GlutaMAX or L-glutamine, 1% nonessential amino acids (all Thermo Fisher Scientific), and 0.1 mM β-mercaptoethanol (Merck) to initiate differentiation. At day 6, medium was changed to EC medium containing 99.5% human endothelial serum-free medium and 0.5% B-27 (all Thermo Fisher Scientific) supplemented with 20 ng/ml hbFGF and 10 µM retinoic acid [26]. At day 8 of differentiation, 1 × 10^6^ cells/cm^2^ were seeded onto collagen IV/fibronectin-coated inserts (0.4 μm pore size, transparent, 24-well format; Greiner Bio-One), 1 mg/ml collagen IV, and 0.5 mg/ml fibronectin (both Merck) in deionized water.

### Generation of PCs

PCs were generated from hiPSCs as recently described with minor modifications [28]. HiPSCs were seeded at day -2 as single cells at a density of 2.5 × 10^4^ cells/cm^2^ on Matrigel-coated plates in mTeSR1 supplemented with 10 µM Y-27632. Medium was replaced by mTeSR1 the next day. To induce differentiation at day 0, medium was changed to mesodermal induction medium consisting of B(P)EL supplemented with 25 ng/ml activin A (Peprotech), 30 ng/ml BMP4 (Thermo Fisher Scientific), 50 ng/ml VEGF (NEB), and 1.5 µM CHIR99021 (Biomol). B(P)EL consisted of 43.8% IMDM with GlutaMAX, 46.3% Ham’s F-12 Nutrient Mix with GlutaMAX, 5% PFHM-II, 1% lipids, 0.1% ITS-X (all from Thermo Fisher Scientific), 2% of 10% BSA (NeoFroxx) in IMDM, 0.3% α-monothioglycerol, and 1% AA2P (both from Sigma Aldrich). Medium was changed daily. From day 3 until day 10 of differentiation, cells were treated with vascular specification medium consisting of B(P)EL supplemented with 50 ng/ml VEGF and 10 µM SB 431542 (Tocris). At day 10, cells were separated by Accutase for 7 min at 37 °C for purification of PCs, which was achieved by removal of CD31-positive cells using the anti-CD31 MicroBead Kit (Miltenyi Biotec) for magnetic cell separation. The negative fraction was collected and seeded at a density of 1 × 10^5^ cells/cm^2^ on 0.1% gelatin-coated (Serva) cell culture surfaces. PCs were treated with EGM-2 medium (Lonza) until an 80% confluence was reached, followed by 3 days culture in PC-induction medium consisting of DMEM, 10% FCS (Bio & Sell), 2 ng/ml TGFβ (Prospec), and 4 ng/ml PDGF-BB (Peprotech). Thereafter, cells were cultured in pericyte medium (ScienCell) by changing the medium every other day and passaging at 80% confluence at a split ratio of 1:2−1:3.

### Generation of MGCs

MGCs were generated from hiPSCs as recently described with minor modifications [24, 29, 30]. HiPSCs were passaged at day -1 as small colonies. Cells were cultivated under hypoxic conditions (5% O_2_, 5% CO_2_, 90% N_2_) from day 0 until day 9. At day 0, microglia progenitors were induced within hiPSCs using mTeSR1 medium supplemented with 80 ng/ml BMP4. At day 4, cells were cultivated in StemPro^TM^-34 SFM supplemented with 2 mM GlutaMAX (all Thermo Fisher Scientific), 80 ng/ml VEGFB, 25 ng/ml hbFGF, and 100 ng/ml SCF. At day 6, growth factors were changed to 50 ng/ml SCF, 50 ng/ml IL-3, 50 ng/ml M-CSF, 50 ng/ml FLT3-L, and 5 ng/ml TPO. At day 12, cell growth factors were changed to 50 ng/ml FLT3-L, 50 ng/ml GM-CSF, and 50 ng/ml M-CSF (all growth factors from Peprotech). Media was changed every other day until day 24. Thereafter, cells were cultured in NGD medium by changing the medium every other day and passaging at 80% confluence at a split ratio of 1:2−1:3.

### Generation of NSCs

NSCs were generated from hiPSCs as recently described with minor modifications [31]. HiPSCs were dissociated with Accutase and seeded onto Matrigel-coated plates at a cell density of 15–25 × 10^3^ cells/cm^2^ in mTeSR1 supplemented with 10 µM Y-27632. When cells reached 15–20% confluence, the medium was switched to PSC Neural Induction Medium (NIM; Thermo Fisher Scientific). NIM medium was changed daily until the generation of primitive NSCs at day 7. Subsequently, NSCs were dissociated with Accutase and reseeded onto Matrigel-coated plates at a cell density of 50 × 10^3^ cells/cm^2^ and placed in neural expansion medium (NEM) consisting of 50% NIM and 50% Advanced DMEM/F12 (Thermo Fisher Scientific), and 10 µM Y-27632. Thereafter, cells were maintained in NEM by changing the medium every other day and passaging at 80% confluence at a split ratio of 1:3−1:6. From passage 3 onwards, NSC lines were cryopreserved in Bambanker (Nippon Genetics) or used for further experiments.

### Generation of ACs

NSCs were generated as outlined earlier and then differentiated into ACs as recently described with minor modifications [32]. For AC differentiation 2 × 10^4^ NSCs/cm^2^ were seeded on Matrigel in AC differentiation medium consisting of 25% DMEM, 25% Ham’s F12 nutrient mix, and 50% Neurobasal supplemented with 0.5X N-2 supplement, 0.5X B-27 supplement without vitamin A, 2 mM GlutaMAX, 10 ng/ml hbFGF, and 10 ng/ml EGF (Peprotech). At day 2, the AC differentiation medium was supplemented with 1X N-2 supplement, 4% FCS (Lonza), and 10 ng/ml CNTF (Peprotech). At day 16, CNTF was replaced by 0.5 mM dBcAMP (Sigma-Aldrich). At day 23, medium was supplemented with 1X N-2 and 4% FCS. At day 30, cells were passaged onto T25 flasks coated with 10 µg/ml poly-L-lysin and cultured in AC medium (both Pelo Biotech). ACs were purified by magnetic cell separation using the anti-GLAST (ACSA-1) MicroBead Kit (Miltenyi Biotec). Positively selected ACs were maintained in AC medium by changing the medium every other day and passaging at 90% confluence at a split ratio of 1:4−1:8.

### High-throughput multiplex qPCR

BCECs with transendothelial electrical resistance (TEER) values of 900–2300 Ω*cm^2^ and a permeability coefficient of sodium fluorescein (PC_NaF_) range of 0.1-1.0 were utilized for a high-throughput multiplex quantitative real-time PCR to evaluate 90 mRNA transcripts important for BBB integrity and functionality as well as 4 internal controls (Additional file 1: Table S1). Total RNA was isolated using a NucleoSpin™ RNA Kit (Macherey-Nagel) according to the manufacturer’s protocol. A total of 250 ng RNA per sample was transcribed into a volume of 20 µl cDNA using the High Capacity cDNA Reverse Transcriptase Kit (Thermo Fisher Scientific) according to the manufacturer’s instructions. For preamplification of the samples, Qiagen Mastermix and HotStarTaq Plus Polymerase (Qiagen) combined with 10X concentration of gene targeting primers was used. High-throughput qPCR was performed using the Biomark™ System (Fluidigm™), including an IFC Controller HX and 96.96 Dynamic Array™ IFCs by running the following program: 15 min at 95 °C, 18 cycles of 40 s at 95 °C, 40 s at 60 °C, 80 °C at 72 °C, and 7 min at 72 °C. The extreme Studentized deviate method (Grubbs’ test) was applied to threshold cycle (Ct) values to detect outliers. Ct values were normalized to the geometric mean of four internal control genes peptidylprolyl isomerase A (PPIA), actin beta (ACTB), glyceraldehyde-3-phosphate dehydrogenase (GAPDH), and beta-2-microglobulin (B2M). Relative quantification was calculated according to the comparative 2^−ΔΔCt^ method [33]. Statistical significance (p ≤ 0.05) was calculated with the Student’s t-test using normalized Ct values.

### Telomere length analysis

For telomere length analysis, monochromatic multiplex qPCR was performed to measure the telomere/single copy gene ratio as previously described [34]. Genomic DNA was extracted from hiPSCs as well as differentiated ACs, BCECs, NSCs, and PCs using the DNeasy™ Blood & Tissue Kit (Qiagen) by following the manufacturer’s protocol. The analysis was performed with 1X HOT FIREPol™ EvaGreen™ qPCR Mix (Solis Biodyne), 900 nM of each telomere primer (forward primer, 5’-ACACTAAGGTTTGGGTTTGGGTTTGGGTTTGGGTTAGTGT-3’; reverse primer, 5’-TGTTAGGTATCCCTATCCCTATCCCTATCCCTATCCCTAACA-3’), 500 nM of each reference primer for albumin (ALB) (forward primer, 5’-CGGCGGCGGGCGGCGCGGGCTGGGCGGaaatgctgcacagaatccttg-3’; reverse primer, 5’GCCCGGCCCGCCGCGCCCGTCCCGCCGgaaaagcatggtcgcctgtt-3’), and 20 ng genomic DNA using the CFX Connect™ Real-Time Detection System (BioRad). Measurements were performed in technical triplicates of three independent differentiations for each cell type (mean ± SEM). Statistical significance (p ≤ 0.05) was calculated with the unpaired Welch’s t-test using normalized Ct values.

### Short tandem repeat (STR) analysis

STR analysis was performed to demonstrate that hiPSCs and the isogenic brain cells (BCECs, PCs, ACs, NSCs) derived from the same donor. DNA isolation was performed using the DNeasy Blood & Tissue Kit (Qiagen) according to the manufacturer’s instructions. DNA was amplified with the PowerPlex™ ESX 17 Fast System (Promega) for 30 cycles. This system allows co-amplification of 16 autosomal STR loci and amelogenin (for gender determination). Amplification products were separated using an Applied Biosystems™ 3500 Genetic Analyzer.

### Flow cytometry

Cells were harvested using Accutase or TrypLE™ (both Thermo Fisher Scientific), a single cell suspension was prepared, and cell numbers were determined. 1–2 × 10^5^ cells were fixed with BD Cytofix™ fixation buffer (BD Biosciences) for 20 min or directly stained using conjugated antibodies and the corresponding isotype controls. If non-conjugated antibodies were used, cells were additionally incubated in a blocking solution (5% donkey serum in PBS) for 30 min. Optionally, for cell surface staining, cells were incubated with 80 µl wash buffer and 1 µl FcR blocking reagent (Miltenyi Biotec) for 20 min. Prior to staining, cells were washed twice with wash buffer (0.5% BSA, 1% FCS, and 2 mM EDTA in PBS) or with Cell Staining Buffer (BioLegend). Antibody incubation was performed for 10–30 min on ice in the dark. The staining with unconjugated primary antibodies was followed by two washing steps with wash buffer or Cell Staining Buffer and incubation with the appropriate secondary antibody for 30 min on ice. The following antibodies were used: Mouse IgG_1_ anti-human alanyl aminopeptidase, membrane (ANPEP/CD13) APC (1:10; #557454, BD Biosciences), Mouse IgG_1_ isotype control APC (1:10; #554681, BD Biosciences), Rabbit IgG anti-human platelet-derived growth factor receptor beta (PDGFRB/CD140B; 1:50; #ab32570, Abcam), Donkey IgG anti-rabbit Alexa Fluor 488 (1:400; #A-21206, Thermo Fisher Scientific), Mouse IgG_2a_ anti-human excitatory amino acid transporter 1 (EAAT1/SLC1A3/GLAST) PE (1:50; #130-118-483, Miltenyi Biotec), Mouse IgG_2a_ isotype control PE (1:50; #130-113-834, Miltenyi Biotec), Human IgG_1_ anti-human protein thyrosin phosphatase receptor type C (PTPRC/CD45) FITC (1:200; #130-110-769, Miltenyi Biotec), Human IgG_1_ isotype control FITC (1:200; #130-113-437, Miltenyi Biotec), Human IgG_1_ anti-human paired box 6 (PAX6) PE (1:10; #130-123-311, Miltenyi Biotec), Human IgG_1_ isotype control PE (1:10; #130-104-613, Miltenyi Biotec), Mouse IgG_2a_ anti-human SRY-box transcription factor 2 (SOX2) Alexa Fluor 647 (1:10; #560302, BD Biosciences), Mouse IgG_3_ isotype control Alexa Fluor 647 (1:10; #560803, BD Biosciences), Human IgG_1_ anti-human SOX1 FITC (1:50; #130-111-157, Miltenyi Biotec), and Human IgG_1_ isotype control FITC (1:10; #130-113-449, Miltenyi Biotec). Stained cells were washed 3X with wash buffer or Cell Staining Buffer and then resuspended in 200 µl washing buffer or 300 µl Cell Staining Buffer. The labeled cells were analyzed using the BD Accuri™ C6, BD FACS Canto™, or the BD FACS Calibur™ flow cytometer (all from BD Biosciences). Cells were checked for viability using propidium iodide or 7-AAD to indicate loss of membrane integrity.

### Immunofluorescence analysis

Cells were fixed using 4% paraformaldehyde for 10–15 min. After washing with PBS, cells were permeabilized with 0.1–0.2% Triton™ X-100 for 5–15 min or a combination of 0.1% Triton X-100 and 1% horse serum for 30 min. After additional washing steps, cells were incubated for 20–30 min with blocking solution containing either 3% BSA and 0.1% Tween, 3.5% horse serum only or 5% donkey serum, 0.02% saponin, and 0.1% Triton X-100. The respective primary antibodies were diluted in blocking solution and applied to the cells overnight at 4 °C. The following antibodies and dilutions were used: Mouse IgG_2a_ anti-human actin alpha 2, smooth muscle (ACTA2/αSMA; 1:100; #ab7817, Abcam), Goat IgG anti-human allograft inflammatory factor 1 (AIF1/IBA1; 1:200; #NB100-1028, BioTechne), Rat IgG_2b_ anti-human CD45 (1:200; #NB100-77417, BioTechne), Rabbit IgG anti-human claudin 5 (CLDN5; 1:100; #ab15106, Abcam), Mouse IgG_2a_ anti-human EAAT1/ GLAST (1:100; #sc-515839, Santa Cruz Biotechnology), Mouse IgG_2a_ anti-human EAAT2 (1:100; #sc-365634, Santa Cruz Biotechnology), Rabbit IgG anti-human solute carrier family 2 member 1 (SLC2A1/GLUT1; 1:200; #ab115730, Abcam), Mouse IgG_1_ anti-human nestin (NES; 1:1000; #60091, Stemcell Technologies), Mouse IgG_1_ anti-human chondroitin sulfate proteoglycan 4 (CSPG4/NG2; 1:100; # 83508, Abcam), Mouse IgG_1_ anti-human occludin (OCLN; 1:200; #331500, Thermo Fisher Scientific), Rabbit IgG anti-human PAX6 (1:200; #ab5790, Abcam), Rabbit IgG anti-human SOX1 (1:200; #ab22572 Abcam), and Rabbit IgG anti-human tight junction protein 1 (TJP1/ZO1; 1:400; #21773-1-AP, Proteintech). After washing 3X with PBS, the cells were incubated for 1–2 h at room temperature in the dark with the appropriate secondary antibodies (1:500; Alexa Fluor 488 or 555 or 647, all from Thermo Fisher Scientific) diluted in blocking solution, and washed. Nuclear staining was performed with 1–5 µg/ml Hoechst 33258 or 33342 (both Thermo Fisher Scientific) and cells were imaged directly or samples on glass cover slips were sealed with Dako™ fluorescence mounting medium (Agilent Technologies) prior to imaging. Alternatively, cells were covered with Fluoromount-G™ containing DAPI (eBioscience), and in this case the staining step with Hoechst was omitted. Fluorescent images were acquired using the Operetta CLS High Content Imaging System (PerkinElmer) or the BZ-9000 BIOREVO System (Keyence).

### Measurement of TEER and sodium fluorescein (NaF) permeability

The resistance value was measured using the Millicell ERS-2 (Millipore) and electrode type STX01 (World Precision Instruments). Prior to usage, the electrode was disinfected in 70% ethanol for 15 min and equilibrated in EC medium for the same amount of time. Electrical resistance (Ω) was measured at day 10 of differentiation on BCECs in mono- or co-cultures in EC medium. Measurements were performed at 37 °C on a heated platform exactly 40 min after medium change. To determine the TEER values (Ω*cm^2^), the electrical resistance of collagen IV/fibronectin-coated inserts was first determined as the mean of three measurements (blank value). Then, the electrical resistance of the BCECs was determined as the mean of three measurements, the blank value was subtracted, and this was multiplied by the culture area of the inserts (cm^2^). Measurements were performed for at least three independent BCEC differentiations (mean ± SEM). TEER values were confirmed to peak on day 10.

Measurement of NaF permeability was performed on a rocking shaker at 100 rpm, 37 °C, 95% humidity, and 5% CO_2_. 200 µl of EC medium containing 10 µM NaF (0.33 kDa) was applied to the apical (upper) side of inserts cultured with BCECs. 850 µl of EC medium was applied to the basolateral (lower) side of the insert on the well plate. After 60 min, samples were taken from both compartments and fluorescence intensity was measured using a fluorescence reader (TECAN Infinite M200, TECAN Infinite M1000 Pro, TECAN Infinite 200 PRO) with the following settings: absorption 490 nm, emission 525 nm. Measurements were performed for at least three independent BCEC differentiations (mean ± SEM). Fluorescence intensities were used to calculate PC_NaF_ according to the clearance principle as previously published [35].

TEER and PC_NaF_ values were normally distributed and analyzed for significance using the Student’s t-test (p ≤ 0.05). Data was displayed by box and whisker plots showing median, interquartile range, min, and max. In addition, absolute TEER and PC_NaF_ values are provided in Additional file 1: Table S3.

### Statistical analyses

Depending on the data analyzed, differences between experimental groups were determined as indicated in the respective sections using GraphPad Prism Software (GraphPad Software, Inc.).

## Results

### An isogenic hiPSC-derived model of the NVU contains cell types with characteristic marker expression

To mimic the NVU in vitro, we used hiPSCs generated from blood of a LOAD patient (LOAD NVU model) and of a healthy elderly control subject (CON NVU model) to obtain isogenic ACs, BCECs, MGCs, NSCs, and PCs. We performed STR analysis for the hiPSCs and their derived cells to confirm common ancestry (Additional file 1: Table S2).

Since the healthy subject and the LOAD patient had reached a comparable age at the time of sample collection, we wanted to determine whether an aging marker, telomere length, was similar between the two hiPSC populations. Moderately increasing shortening of telomeres is associated with aging while increased shortening of telomeres beyond physiological levels is associated with the onset of LOAD [36]. We found no changes in hiPSCs and PCs but a LOAD-dependent decrease in telomere length in ACs, BCECs, and NSCs, thus integrating a disease-related molecular phenotype for LOAD in our isogenic in vitro model of the NVU (Additional file 1: Fig. S1).

**Fig. 1:**
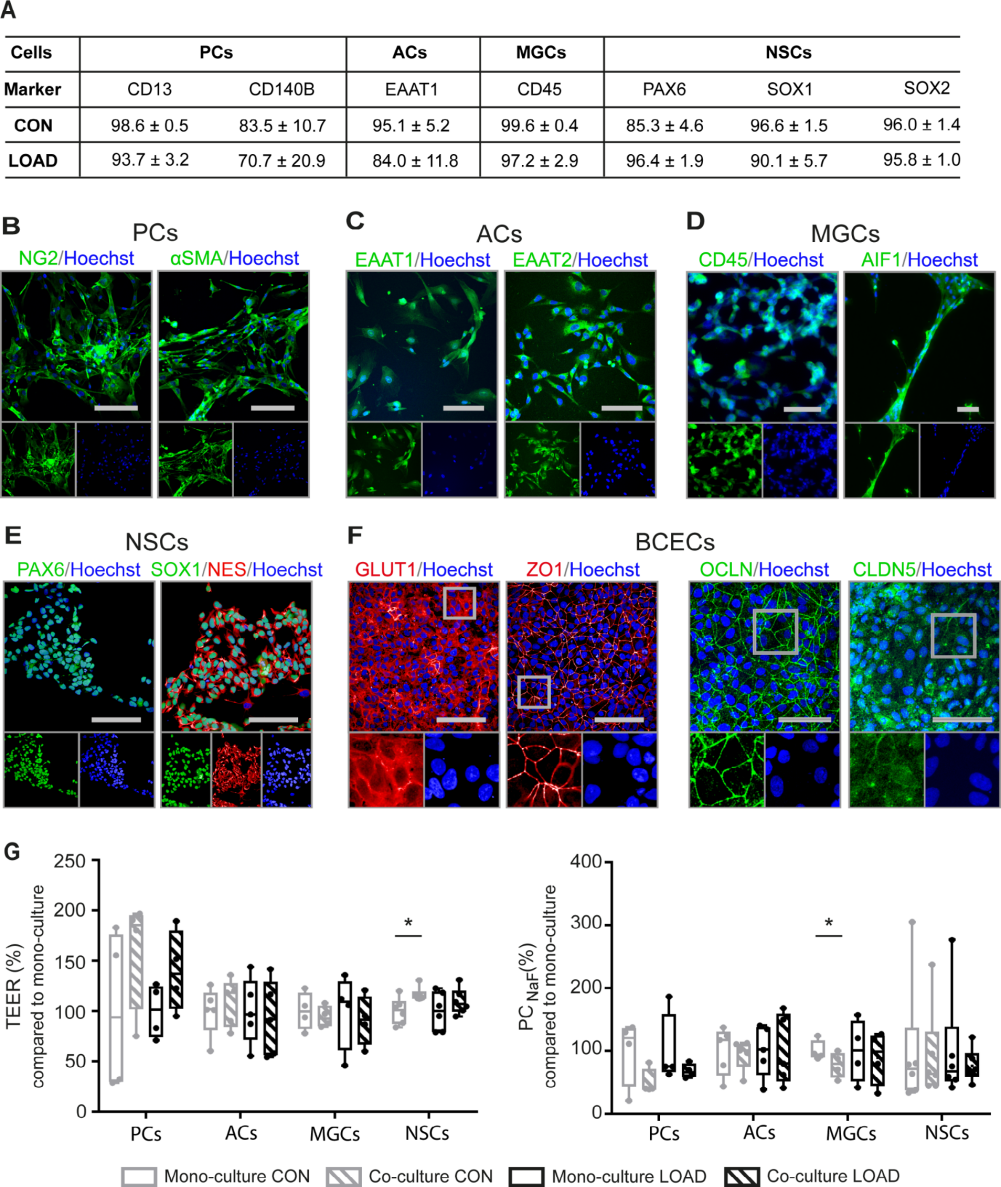
Expression and localization of marker proteins in hiPSC-derived brain cell types of the NVU. HiPSCs were derived from a late-onset Alzheimer disease patient (LOAD NVU model) and a healthy elderly control subject (CON NVU model). (**A**) Flow cytometry confirmed high expression of cell type-specific phenotypic markers in pericytes (PCs), astrocytes (ACs), neural stem cells (NSCs; all mean ± SD; n ≥ 3), and microglia-like cells (MGCs; mean ± SD; n = 2). Representative immunofluorescence images confirmed protein expression of (**B**) NG2 and αSMA in PCs, scale bar 200 μm, (**C**) EAAT1 and EAAT2 in ACs, scale bar 100 μm, (**D**) CD45 and AIF1 in MGCs, scale bar 100 μm, (**E**) PAX6, SOX1, and NES in NSCs, scale bar 100 μm, and (**F**) GLUT1/SLC2A1, ZO1, OCLN, and CLDN5 in BCECs, scale bar 100 μm. (**G**) Measurement of transendothelial electrical resistance (TEER) and sodium fluorescein (NaF) permeability to analyze the integrity of the blood-brain barrier in CON and LOAD. TEER (*left*) and permeability coefficient (PC_NaF_) (*right*) values are shown for mono- and co-cultures consisting of BCECs with or without PCs, ACs, MGCs, or NSCs. Box (median and lower/upper quartile) and whisker (minimum/maximum) plots display n = 4–6 independent biological assays (no outliers), one-tailed t-test, *p < 0.05

Flow cytometry was applied to confirm the purity of the brain cell types used (Fig. 1A). PCs in passages 2 to 4 showed high expression of brain PC markers CD13 and CD140B/PDGFRβ with more than 90% and 70% positive cells, respectively. Flow cytometry analysis of the ACs showed that the excitatory amino acid transporter EAAT1 was present in > 80% of the cells. The presence of CD45 was detected in > 95% of MGCs, confirming their microglia-like identity. Assessment of early neuronal markers showed a high purity of NSCs. More than 85% of the cells expressed PAX6, > 90% expressed SOX1, and > 95% expressed SOX2.

The expression and localization of phenotypic marker proteins in hiPSC-derived brain cell types of the NVU were investigated by immunofluorescence analysis in the LOAD NVU model (Fig. 1) and the CON NVU model (Additional file 1: Fig. S2). In PCs, the characteristic protein neuroglial antigen 2 (NG2) was expressed on the cell surface and actin, aortic smooth muscle (αSMA) was detected in the cytoplasm (Fig. 1B). ACs expressed EAAT1 and EAAT2 (Fig. 1C) in nucleoli and vesicle-like structures in the cytoplasm. The microglia-like phenotype was confirmed by the presence of CD45 and AIF1 in the cytoplasm of MGCs (Fig. 1D). In NSCs, the transcription factors PAX6 and SOX1 were detected in the nucleus and the neuronal intermediate filament NES was detected in the cytoplasm (Fig. 1E). BCECs expressed the glucose transporter member 1 (GLUT1/SLC2A1) predominately at the cell membrane. The TJ-specific proteins zonula occludens 1 (ZO1/TJP1), OCLN, and CLDN5 formed a uniform network (Fig. 1F).

Altogether, different brain cell types were derived with comparable efficiency and degree of differentiation from hiPSCs obtained from a healthy subject and a LOAD patient.

### Co-cultures tighten the BBB in CON but not in LOAD

To study the influence of different co-cultures consisting of BCECs and PCs, ACs, MGCs, or NSCs on the integrity of the BBB in CON and LOAD models, TEER and NaF permeability were measured (Fig. 1G, raw data in Additional file 1: Table S3). The basolateral co-cultured brain cell types do not have direct cell contact with BCECs in the apical compartment but they modulate their integrity and functionality by secretion of soluble factors through membrane pores in the inserts [11]. In the CON NVU model, barrier integrity of BCECs was significantly increased by NSCs (p = 0.02; TEER = 2674 ± 670 Ω*cm^2^) compared to BCEC mono-cultures (TEER = 2299 ± 123 Ω*cm^2^) (Fig. 1G). The supporting effect was suppressed in the LOAD NVU model. Co-cultures with ACs, MGCs, or PCs did not significantly alter TEER values in CON or LOAD.

To characterize paracellular transport, NaF permeability of the BCECs was measured. We observed significant changes of PC_NaF_ values in the CON NVU model. MGCs reduced NaF permeability (p = 0.05; PC_NaF_ = 0.840 ± 0.111 μm/min) compared to BCEC mono-cultures (PC_NaF_ = F1.080 ± 0.088 μm/min) (Fig. 1G). Significant changes of barrier integrity as monitored by TEER and NaF permeability was not observed between CON and LOAD models in BCECs alone and BCECs co-cultured with PCs, ACs, MGCs, or NSCs (Fig. 1G).

### BCECs have LOAD-specific gene expression profiles in response to all co-cultures

High-throughput multiplex qRT-PCR that included 90 BBB markers was used to analyze changes in gene expression in CON and LOAD BCECs in response to co-cultured isogenic PCs, ACs, MGCs, or NSCs.

**Fig. 2:**
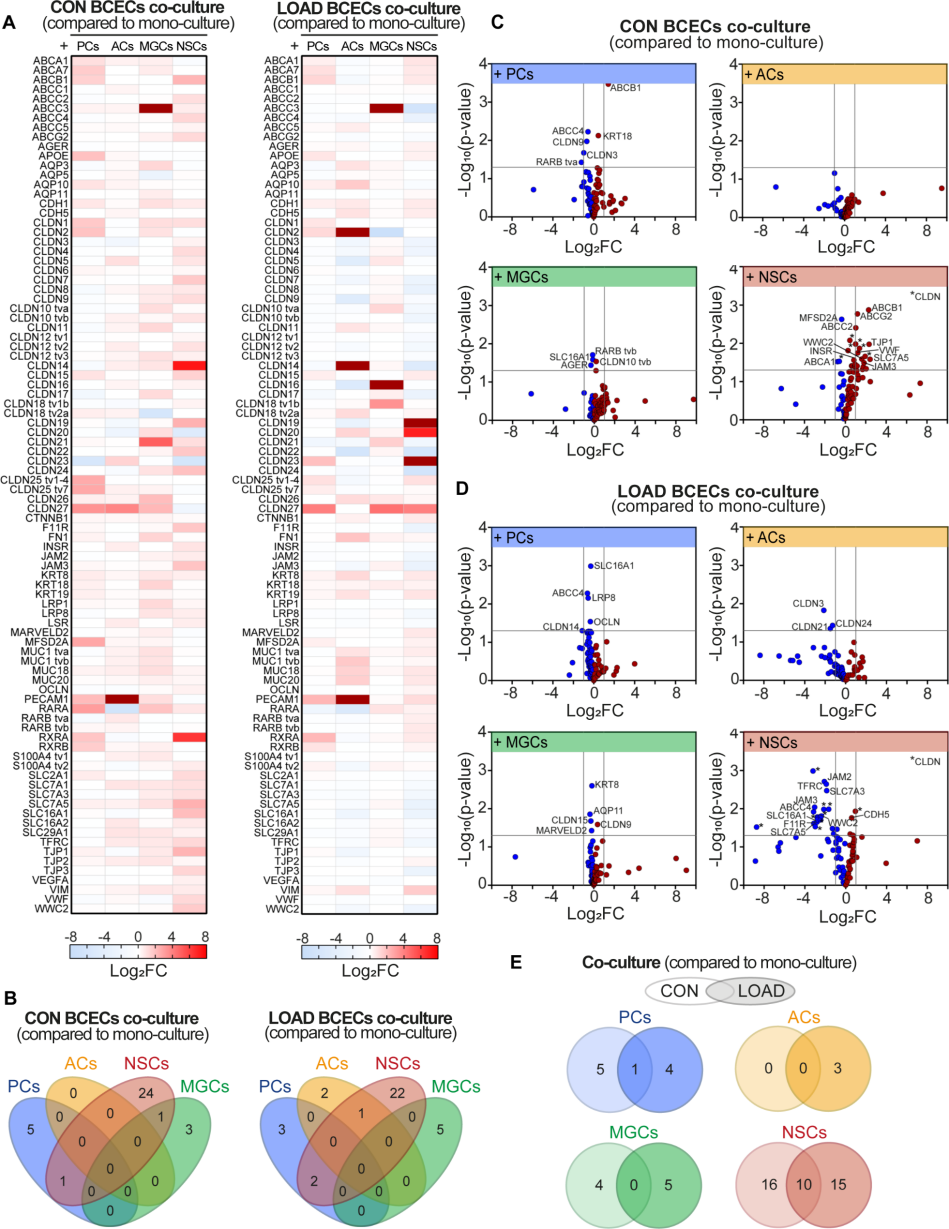
Transcriptional alterations in LOAD or CON BCECs in mono- and co-culture with different NVU cell types. Comparison of mono- and co-cultures in the CON and LOAD NVU models by high-throughput multiplex qRT-PCR analysis of BBB markers expressed in BCECs (n = 3‑5 independent differentiations). (**A**) HiPSC‑derived CON and LOAD NVU models are displayed separately. The values of the logarithm of fold change (Log_2_FC) for each transcript is plotted on a heat map indicating up-regulated (*red*) and down-regulated (*blue*) transcripts. (**B**) Venn diagram showing the number of significantly regulated transcripts (p ≤ 0.05) shared between co-cultures with different brain cell types. CON and LOAD NVU models are displayed separately. (**C**) Volcano blot displaying Log_2_FC and p-values for each transcript in the CON NVU model (comparison of CON mono‑ and co‑cultures). Significantly up- (*red*) and down-regulated (*blue*) transcripts are shown. (**D**) Volcano blot displaying Log_2_FC and p-values for each transcript in the LOAD NVU model. (**E**) Venn diagram showing the number of significantly regulated transcripts shared between CON and LOAD co-cultures. BCECs, brain capillary endothelial-like cells; ACs, astrocytes; MGCs, microglia-like cells; NSCs, neural stem cells; PCs, pericytes, tv, transcript variant

First, CON and LOAD NVU models were studied separately in order to investigate the influence of co-cultured brain cells on the mRNA expression of BBB markers in the related isogenic BCECs (Fig. 2). A wide range of transcripts was up- or down-regulated by addition of co-culture cells. The CON NVU model with co-cultures of BCECs and PCs, ACs, MGCs, and NSCs showed more up-regulated transcripts (Log_2_FC > 0) and less down-regulated transcripts (Log_2_FC < 0) compared with the LOAD NVU model (Fig. 2A).

No single transcript was significantly differentially regulated in all co-cultures in CON or LOAD BCECs (Fig. 2B). The number of significantly altered transcripts (p ≤ 0.05) that were observed in CON BCECs co-cultured with isogenic cells were ACs = 0, NSCs = 26, MGCs = 4, and PCs = 6; and LOAD BCECs co-cultured with isogenic cells were ACs = 3, NSCs = 25, MGCs = 5, and PCs = 5 (Fig. 2B). Observed effects were most prominent in CON and LOAD BCECs co-cultured with NSCs (Fig. 2B-E). CON and LOAD BCECs showed different patterns of up- and down-regulated transcripts dependent on the co-culture composition. In CON BCECs, there was a predominance of downregulated transcripts in co-cultures with PCs and MGCs and a predominance of upregulated transcripts with NSCs (Fig. 2C). In LOAD BCECs, we found a predominance of down-regulated transcripts in all co-cultures (Fig. 2D). There were no genes that were significantly regulated in both CON and LOAD BCECs co-cultured with isogenic ACs and MGCs, but for NSCs these genes included CLDN7, CLDN12 tv2, F11R, JAM3, SLC7A1, SLC7A3, INSR, SLC7A5, SLC16A1, and WWC2 as well as ABCC4 for PCs (Fig. 2E). Again, co-cultures with NSCs had the strongest impact on BBB markers in BCECs.

**Fig. 3:**
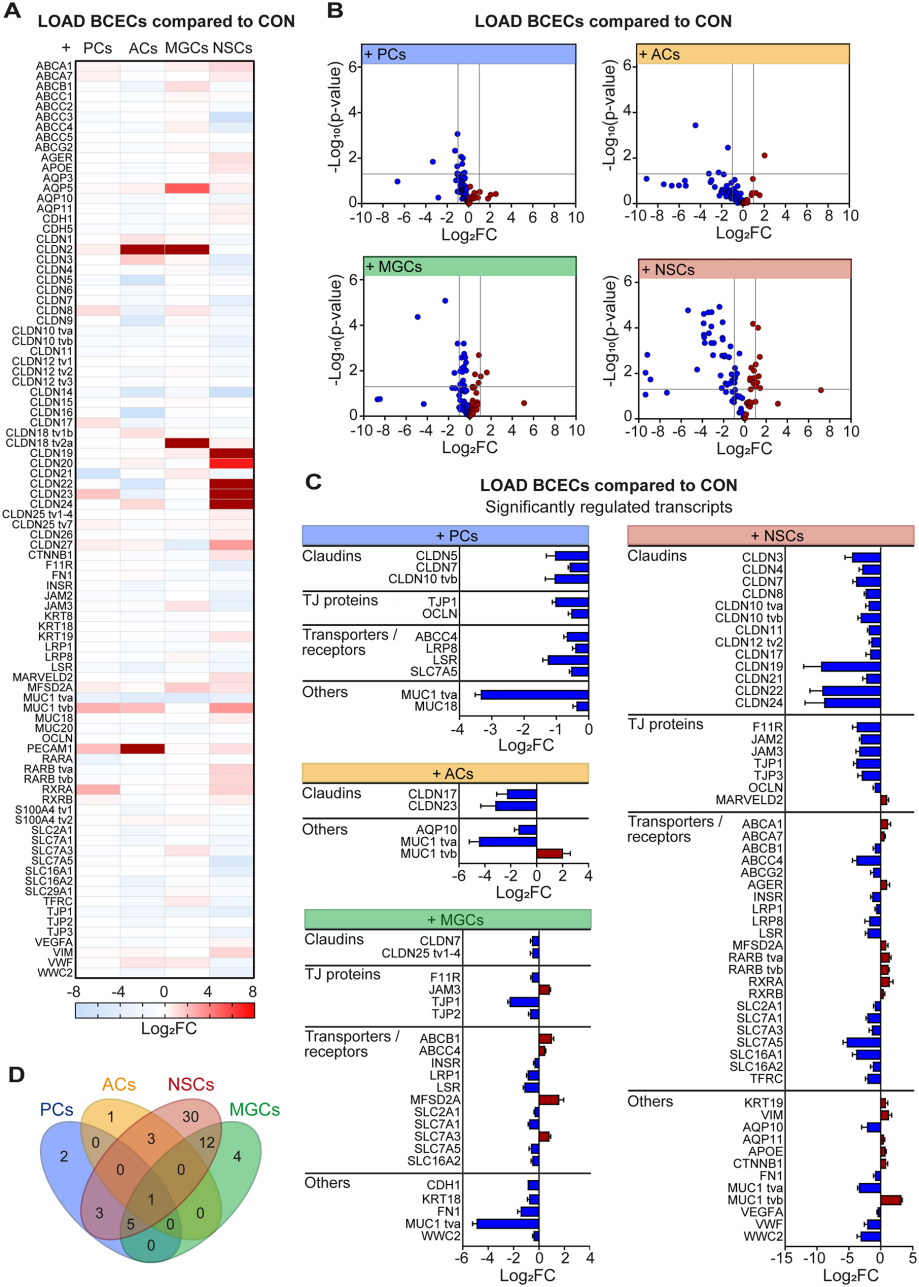
Transcriptional changes of LOAD BCECs co-cultured with isogenic cell types. Comparison of control (CON) and late-onset Alzheimer disease (LOAD) neurovascular unit models by high-throughput multiplex qRT-PCR analysis of blood-brain barrier markers expressed in BCECs (n = 35 independent differentiations). (**A**) The value of the logarithm of fold change (Log_2_FC) for each transcript is plotted on a heat map indicating up- (*red*) and down-regulated (*blue*) transcripts (Log_2_FC > 0 and < 0, respectively). (**B**) Volcano plot displaying Log_2_FC and -Log_10_ (p-values) for each transcript. The Log_2_FC = -1/1 and the p = 0.05 are indicated (*grey lines*). (**C**) List of significantly regulated blood-brain barrier markers (mean + SEM; Student’s t-test, p ≤ 0.05). (**D**) Venn diagram illustrating the number significantly regulated transcripts shared between different brain cell types. BCECs, brain capillary endothelial-like cells; ACs, astrocytes; MGCs, microglia-like cells; NSCs, neural stem cells; PCs, pericytes; TJ, tight junction; tv, transcript variant

Second, we studied differences between the CON and LOAD NVU model to investigate the influence of LOAD on the mRNA expression of BBB markers in the related isogenic BCECs (Fig. 3). Our analysis revealed transcriptional changes in LOAD BCECs compared with CON BCECs. The heat map of Log_2_FC values shows 90 BBB markers in LOAD BCECs compared to CON BCECs (Fig. 3A). In individual co-cultures, 90 BBB markers were detected, and statistical analysis could be performed for 84 to 87 of the BBB markers in LOAD and CON BCECs (ACs: 87; MGCs: 88; NSCs: 87; PCs: 85). The subtracted markers were not detected in each replicate or contained outliers. We found up- and down-regulated transcripts in BCECs co-cultured with ACs (3 and 37, 3% and 42%), MGCs (4 and 11, 5% and 13%), NSCs (10 and 39, 12% and 45%), and PCs (4 and 10, 5% and 12%) for Log_2_FC > 1 and Log_2_FC < -1, respectively. Accordingly, a large number of BBB markers were in the range between Log_2_FC of -1 and 1 (ACs: 54%; MGCs: 83%; NSCs: 44%; PCs: 84%). The volcano plot shows that not all transcripts with Log_2_FC below − 1 or above 1 were found to be significantly regulated (Fig. 3B). The percentage of significantly changed transcripts varied depending on the co-cultured cell type (ACs = 6%; PCs = 13%; MGCs = 25%; NSCs = 62%).

The transcriptional profiles of BCECs differed between the CON and LOAD NVU models depending on which brain cell type was included in the co-culture. For example, PCs down-regulated claudins (CLDN5, -7, -10 tvb), TJ transcripts (TJP1, OCLN), transporters and receptors (ABCC4, LRP8, LSR, SLC7A5), and mucins (MUC1 tva, MUC18) in LOAD BCECs (Fig. 3C). ACs decreased the transcription of claudins (CLDN17, -23) in LOAD BCECs compared to CON BCECs. Mucins were altered with transcript variant a (tva) of MUC1 being reduced and tvb being increased (Fig. 3C). MGCs altered the transcription of claudins (CLDN7, -25 tv1-4) and other TJ (F11R, JAM3, TJP1, TJP2) and adherens junction makers (AJs; CDH1), transporters and receptors (ABCB1, ABCC4, INSR, LRP1, LSR, MFSD2A, SLC2A1, SLC7A1, SLC7A3, SLC7A5, SLC16A2), and other factors (KRT18, FN1, MUC1 tva, WWC2) in LOAD BCECs (Fig. 3C).

NSCs had the strongest effect on transcriptional changes in LOAD BCECs (Fig. 3C) including increased, but mainly decreased expression of numerous claudins (CLDN3, -4, -7, -8, -10, -11, -12 tv2, -17, -19, -21, -22, -24) and TJ proteins (F11R, JAM2, JAM3, TJP1, TJP3, OCLN, MARVELD2). There was also a large number of altered transcripts for other molecule classes such as transporters and receptors. Those LOAD BCECs also showed altered transcription of the five ABC transporter genes ABCA1 (cholesterol efflux regulatory protein, CERP), ABCA7, ABCB1 (permeability glycoprotein, Pgp), ABCC4, and ABCG2 (breast cancer resistance protein, BCRP). We also found increased transcription of the six SLC transporters SLC2A1/GLUT1, SLC7A1 (cationic amino acid transporter 1, CAT1), SLC7A3 (CAT3), SLC7A5 (large neutral amino acid transporter 1, LAT1), SLC16A1 (monocarboxylate transporter 1, MCT1, and SLC16A2 (MCT8). Transcripts involved in lipid metabolism were significantly altered including APOE, two APOE receptors (low density lipoprotein receptor-related protein 1, LRP1 and LRP8), and the gene encoding the lipolysis-stimulated lipoprotein receptor (LSR). Two intermediate filaments (KRT19, VIM) were significantly upregulated. The gene encoding the aging marker advanced glycosylation end-product specific receptor (AGER; receptor for advanced glycosylation end-product, RAGE) was increased. Finally, mucins were also altered including two MUC1 transcripts (tva was reduced and tvb increased) (Fig. 3C). Indeed, down-regulation of tva of MUC1 was observed independent of the respective brain cell type and the only significantly, differentially regulated transcript shared by all LOAD NVU models (PCs, p = 0.014; ACs, p = 0.0004; MGCs, p = 0.00004; NSCs, p = 0.00002) (Fig. 3D). Nevertheless, LOAD BCECs co-cultured with NSCs shared twelve significantly regulated transcripts with MGC co-cultures and three with AC and PC co-cultures, respectively. Five significantly regulated transcripts were shared by all three (co-cultures with PCs, MGCs, and NSCs) LOAD NVU models compared to CON NVU models, including modulation of ABCC4 and down-regulation of CLDN7, TJP1, LSR, and SLC7A5 (Fig. 3C, D).

## Discussion

The structural and multicellular interplay of the NVU is crucial for maintenance and proper function of the BBB. A leaky and dysfunctional BBB is linked to many neurodegenerative diseases, including LOAD [5, 37]. We herein generated cells of the NVU in vitro using hiPSCs from a LOAD patient and a healthy elderly control subject to study the individual impact of cells in close local and functional vicinity to BCECs. We investigated the barrier integrity as well as the transcriptional profile of the BCECs. Additionally, the results demonstrated, that hiPSC-derived ACs, BCECs, and NSCs behaved differently with respect to the molecular aging marker telomere length. LOAD ACs, BCECs, and NSCs had shorter telomeres compared to CON cells. This is in accordance with other studies reporting shorter telomeres in leukocytes of LOAD patients [38, 39]. However, telomere length has been described differently in animal and human studies in different cell types of the brain with significant telomere shortening or lengthening observed [40]. Changes in telomeres may be related to accelerated aging mechanisms in age-related diseases [40], suggesting that our model mimics a typical disease characteristic for LOAD.

The purity and identity of hiPSC-derived ACs, BCECs, MGCs, NSCs, and PCs were confirmed by the expression and localization of cell type-specific marker proteins. HiPSC-derived PCs were identified by CD13, CD140B, NG2, and αSMA. Nonetheless, a definitive identification of PCs and their distinction from other mural cells is difficult due to a lack of specific markers [41]. Therefore, hiPSC-derived PCs are considered as mural cells with a PC-like phenotype. Similarly, hiPSC-derived MGCs expressing CD45 and AIF1 are considered as microglia-like cells that are not fully mature but resemble primary fetal and adult microglia cultured in vitro and exhibit characteristic functional properties such as phagocytosis and the release of cytokines [24, 29, 30]. NSCs positive for SOX1, SOX2, PAX6, and NES were successfully generated and allowed efficient differentiation of ACs characterized by EAAT1 and EAAT2 in accordance with recent reports [31, 32].

Time for differentiation, the differentiation efficiency, the degree of differentiation, and the expression of marker proteins were similar between the generated NVU cells derived from CON and LOAD hiPSCs. This observation is in line with previous studies on hiPSC-derived CNS cell types carrying the APOE ε4 risk allele. For example, while APOE isoform-specific alterations in ACs, MGCs, or PCs have been described with respect to inflammation, Aβ-related pathology, and the metabolism of APOE and lipids, abnormalities during the differentiation process have not been reported [7, 42, 43]. *Post mortem* studies on brain tissue of APOE ε4 carriers [44], APOE4 transgenic mice [45], and other BBB co-culture models based on human APOE ε4-carrying hiPSCs [7], however, suggest that additional NVU cells, especially PCs, mediate structural changes and dysregulation of BCECs.

Co-cultures of BCECs with isogenic ACs, MGCs, and PCs did not improve barrier integrity of the endothelial layer in the LOAD NVU model. NSCs were only found to significantly increase the TEER of BCECs in the CON NVU models. This is in contrast to other studies, which reported that co-cultures with stem cell-derived or primary PCs, ACs, and/or neurons resulted in improved barrier integrity, TJ continuity, or optimized transporter functionality [46–51]. Nevertheless, Jamieson et al. suggested that during steady-state conditions, BCECs did not require co-cultures with PCs to achieve TEER values above 1500 Ω*cm^2^ [52]. In stressed or suboptimal conditions, however, indirect co-cultures with hiPSC-derived PCs or conditioned medium increased TEER and stabilized the endothelial layer. In our studies, NaF permeability was significantly decreased in CON BCECs by isogenic MGCs, indicating an increased barrier integrity. The supportive effects observed for NSCs and MGCs were diminished in LOAD NVU models. MGCs with anti-inflammatory phenotype (M2) are known to protect the BBB during inflammation [53, 54], playing a crucial role also in AD. Thus, the loss of the barrier-tightening effect of microglia could be due to less effective microglial support of the BBB in LOAD.

BBB dysfunction and breakdown are associated with neurodegenerative diseases and hiPSC-derived models have recently been used to understand the development and progression of these conditions. However, the majority of studies employing patient-derived hiPSC models have concentrated on rare familial forms of AD, which are typically associated with an early onset (typically diagnosed before the age of 65) [55]. For instance, hiPSC-BCECs obtained from patients carrying mutations in the PSEN1 gene show an altered phenotype and compromised BBB function. This includes the loss of barrier integrity, changes in the expression of junctional proteins and efflux pumps, increased production of radical oxygen species, as well as modifications in the secretion and permeability of Aβ peptides [56–58]. While those models provide valuable insights into how autosomal dominant mutations linked to AD may affect BBB function, they have limitations understanding the features of BBBs in patients who exhibit a late onset of the disease [55]. Sporadic LOAD differs from the familial form in terms of disease progression and genetic features [59, 60]. Yet, only a limited number of studies have explored the effects of the genetic background associated with LOAD on hiPSC-derived brain cell types and the neurovascular unit (NVU). Ding et al. [61] reported that presence of the APOE ε4 allele does not directly lead to BBB dysfunction. Furthermore, TJ expression, efflux transporter activity, and TEER values remained constant when comparing hiPSC-derived BCECs carrying APOE ε4 versus cells harboring the APOE ε2 or APOE ε3 risk allele [61]. In an isogenic model described by Faal and colleagues [62], mesoderm- and neural crest-derived PCs significantly increased endothelial integrity compared to the corresponding brain microvascular endothelial cell mono-culture. Furthermore, BBB models derived from healthy control subjects carrying APOE3/3 displayed increased TEER values compared to the corresponding AD-specific models [62].

In our studies, the BCECs derived from LOAD and CON hiPSCs expressed mRNAs for all screened TJ markers including a broad spectrum of claudins (CLDN1-27) as well as CDH5, JAM1-3, MARVELD2, and OCLN. These findings support the theory that TJ structure at the BBB is highly complex and heterogeneous. Transcriptomic analyses from Berndt et al. on human brain capillaries dissected from human brains also showed expression of claudins, CDH5, TJP1, and OCLN [63].

We observed that many, but not all, claudins were significantly regulated in BCECs, both when comparing mono- to co-cultures and CON to LOAD NVU models. CLDN5 is known to be a dominant TJ protein at the BBB [63, 64] suggesting that loss of this protein can impair BBB integrity. Our study showed decreased CLDN5 gene expression in co-cultures of LOAD BCECs and PCs, but not generally in all co-cultures of the LOAD NVU model. The dominant role of CLDN5, however, has been questioned and might be the result from loss of TJ complexity in in vitro conditions [63]. Although CLDN5 and CLDN12 were reported as key claudins at the BBB, they are also expressed by peripheral ECs. We observed in LOAD BCECs the down-regulation of claudins, which function as paracellular sealing proteins (CLDN3, -4, -5, -11, -19, and OCLN) or paracellular pore-forming proteins (CLDN7, -8, -10, -17, -21). Berndt et al. described a dominant role for the paracellular pore-forming proteins CLDN2, -15, and -17 in human capillaries [63]. In our study, isogenic NSCs affected the largest number of TJ proteins in LOAD BCECs compared to other brain cell types in the LOAD NVU model.

Isogenic NSCs significantly up-regulated MARVELD2 expression in LOAD BCECs, raising the question of the importance of this TJ protein. Daneman and colleagues postulated that the transmembrane markers OCLN and MARVELD2 are highly enriched in brain ECs [63]. MARVELD2 seals TJs at points where three cells meet. The authors suggested that this tripartite sealing adhesion contributes to the extremely tight BBB. A similar function has been suggested for the LSR protein [65], which was reduced in LOAD co-cultures with PCs, MGCs, and NSCs.

TJs and AJs play an important role in regulating the permeability of the BBB. However, the most abundant cytoskeletal structural components are intermediate filaments but their impact on the BBB has only recently been recognized (reviewed in [66]). We found that co-cultures of BCECs with NSCs significantly increased gene expression of keratin 19 (KRT19) and vimentin (VIM) in LOAD BCECs, but the presence of MGCs led to a decrease of KRT18. VIM is the most abundant intermediate filament in microvascular ECs, and forms a desmosome-like complex called complexus adherens when bound to VE-cadherin and thereby provides structural support and tissue integrity [67–72]. In pathological conditions of the NVU, such as those found in neurodegenerative diseases, in addition to reorganization of actin and stress fibers there is a disruption of the VIM network which affects permeability of the BBB [73–75]. Therefore, treatment of BCECs with shear stress could help to better understand whether damage to the BBB in LOAD could serve as a link between impaired blood flow and BBB breakdown in AD [76].

Significant changes in the expression of several mucins were observed in LOAD BCECs. The presence of PCs resulted in down-regulation of MUC18 (melanoma cell adhesion molecule, MCAM) gene expression [77], which is closely related to neuronal and platelet-endothelial cell adhesion molecules NCAM1, NCAM2, and PECAM1, and therefore associated with angiogenesis, cell migration, and cell-cell interaction. MUC18 is expressed in vascular endothelium [78, 79], smooth muscle cells [80], and CNS tissue [81]. Here, MUC18 gene expression was found in hiPSC-derived BCECs. MUC1 tva was found to be significantly decreased in all co-cultures with LOAD BCECs. Apart from its role in cell adhesion, protection, and hydration of epithelial surfaces [82, 83], membrane-bound mucin has also been described as an aging marker [84, 85]. The MUC1 ectodomain can be cleaved by γ-secretase, an enzyme involved in Aβ accumulation, a hallmark of AD [86]. Porowska et al. showed that human umbilical vein ECs were capable of synthesizing and releasing MUC1 and treatment with proinflammatory mediators tumor necrosis factor alpha (TNF-α) and interferon gamma increased MUC1 protein and its incorporation into cell membranes of ECs [87]. In addition, MUC1 is expressed in hematopoietic cells [88, 89], including macrophages [90], and in corneal and pulmonary endothelium [91, 92]. Although MUC1 has not been described in LOAD etiology, it plays a role in the context of chronic diseases associated with immune response and inflammation [93]. Our results show that MUC1 tva was not only down-regulated in all cocultures with LOAD BCECs, but MUC1 tvb was significantly increased in co-cultures with ACs or NSCs. MUC1 tva was reported to be less anti-inflammatory compared to MUC1 tvb in COS-7 monkey kidney fibroblasts when TNFα-induced cytokine responses were examined [94]. This data may be important since mucins regulate signaling pathways for BBB function and integrity, and may influence pathomechanisms relevant to AD.

The exchange of nutrients and metabolic end products at the BBB is tightly regulated by influx transporters (including the SLC superfamily members) and efflux transporters (including members of the ABC superfamily) which facilitate the transport of waste products, endogenous toxins, and xenobiotics into the blood. Alterations in SLC and ABC proteins are involved in BBB breakdown in the context of AD [95–97]. Gene expression data from LOAD BCECs suggest that the presence of isogenic ACs, MGCs, NSCs, and PCs can affect the expression of transporters. For example, the glucose transporter SLC2A1 was reduced in LOAD BCECs by MGCs and NSCs. Decreased protein levels of SLC2A1 were also reported in the brain microvascular endothelium of AD patients [98, 99] and deficits in glucose metabolism are linked to AD pathology [100]. In addition, a significant down-regulation of the amino acid transporter SLC7A5 in LOAD BCECs co-cultured with MGCs, NSCs, and PCs was observed. SLC7A5 plays an important role in the treatment of Parkinson’s disease by facilitating the transport of exogenous L-DOPA [95]. Wittmann and colleagues reported the down-regulation of SLC7A5 at the BBB in response to endotoxin-induced inflammation in rodents [101]. However, Gynther et al. showed that neither inflammation nor AD models based on transgenic mice led to altered function of SLC7A5 [102]. Accordingly, the reduction of SLC7A5 mRNA expression in LOAD BCECs described here requires further investigation on the role of SLC7A5 protein at the NVU.

Other studies have documented a link between reduced expression or diminished activity of a variety of ABC transporters with AD pathology which can result in reduced efflux of harmful substances [5]. Our data showed decreased ABCB1 gene expression in LOAD BCECs co-cultured with NSCs. ABCB1 is associated with AD in terms of reduced expression levels in AD patients, impaired clearance of Aβ at the BBB, and accumulation of Aβ in the brain [103–106].

Receptors involved in lipid transport were found to be differentially regulated in LOAD BCECs co-cultured with different brain cell types. Transcripts of different APOE receptors were reduced in LOAD BCECs co-cultured with PCs, NSCs, and MGCs. Numerous studies that have investigated APOE describe the critical role of lipoprotein receptors and the distribution and transport of lipids at the NVU and in the brain [6]. Moreover, LRP1 mediates endocytosis and transcytosis of multiple ligands across the BBB, among them Aβ. AD patients have lower LRP1 blood levels which is accompanied by reduced brain-to-blood clearance of Aβ [107–110]. LOAD BCECs co-cultured with PCs, MGCs, and NSCs also displayed reduced expression levels of LSR, another lipoprotein receptor of relevance in lipid metabolism and AD. For example, diminished LSR levels in Lsr^+/−^ mice was found to be associated with aberrant cholesterol distribution in the brain and this model was more susceptible to Aβ stress [111].

The expression of the aging marker AGER was induced in LOAD BCECs co-cultured with NSCs. AGER is elevated in micro vessels of human AD hippocampi and promotes re-entry of circulating Aβ into the brain [110, 112, 113]. Taken together, the differential expression of receptors and transporters highlights recent findings regarding the supportive role of specific NVU cells for key functions of the BBB and provides evidence that LOAD-related changes at the BBB may contribute to disease progression.

### Limitations

The observed changes in gene expression of BBB markers imply a change at the protein level but this cannot be predicted with certainty since this correlation is weak. The main purpose of the gene expression analysis was to screen targets which have not been previously investigated for better understanding of LOAD disease mechanisms that specifically lead to impairment of the BBB. Further analyses, including functional and proteomic studies, are therefore required to verify these findings. Furthermore, the generality of effects observed when comparing hiPSC model systems derived from only one LOAD patient and one healthy donor is limited and requires further studies with additional hiPSCs from LOAD patients, healthy elderly subjects, and/or genetically engineered hiPSCs.

Other human brain tissue transcriptomic studies or single cell sequencing approaches have examined larger datasets, but they do not necessarily contain all the targeted BCEC BBB markers studied here [114, 115]. These datasets show differences in the up- and down-regulation of certain transcripts, confirming or contradicting our data. However, there are many different EC types in the human brain that are difficult to distinguish using current tissue or capillary dissection techniques and it is often unclear which cell type (or mixture of cell types) from the human brain this population corresponds to. One advantage of our hiPSC-derived cell models is that they represent pure cell populations of BCECs that mimic microvascular ECs of brain capillaries [116].

Regarding cellular identity, a recent study [117] challenges the hiPSC-derived BCEC model applied here [25, 26], which was further refined, evaluated and widely applied over the last decade, including for the study of disease-associated genetic variants. Lu et al. claim that the latter differentiation protocol does not generate cells of endothelial, but of epithelial origin. Opposed to the findings by Lu et al., we have previously reported an increase in endothelial transcripts (e.g. VWF, CDH5, ABCG2, ABCB1) during the course of differentiation for two independent hiPSC lines (WISCi004-B and ZIPi013-B) and specific staining of key BCEC marker proteins (including CLDN5) after completion of the differentiation [116]. This is in line with other publications in the field [18, 118, 119]. It is well appreciated that hiPSC-based models can show a lack of maturity in expression signatures and function. Therefore, epithelial expression signatures from pluripotent stem cells might be maintained after differentiation, like shown here for CLDN3, CLDN6 and CLDN7, which Lu et al. describes as epithelial markers. Having said this, several studies using human primary BCECs and hiPSC-derived BCECs have been published over the last years reporting the expression of almost all claudins in human BBB in vitro models [119–121]. Of note, the alternative protocol suggested by Lu et al. cannot achieve a high paracellular tightness comparable to the physiological situation in vivo, which is of importance for our LOAD model to reliably test the molecular and functional consequences of disease-associated haplotypes. All our data was generated with hiPSC-derived BCECs, characterized by TEER values around 1000 Ω*cm^2^ on day 10 of differentiation.

There is important evidence that the presented model provides valuable results although the findings should be recapitulated with cell lines from additional donors. Based on the detailed characterization of our hiPSC lines [23], we can assume that the found differences are not due to reduced quality of the utilized hiPSC lines. Based on the available literature, we can also assume that differences between hiPSCs from diseased and healthy individuals are usually much stronger than differences between hiPSCs from either of these two groups. A study by [122] also suggests that isogenic approaches, as in this study, reduce variability. In addition, the variability of hiPSC-based models can be reduced if endpoints are defined for characterization [123] as this was performed in the presenting work.

## Conclusion

In summary, different brain cell types of the NVU were found to have an impact on BCECs, and this in turn may lead to greater insights into the regulation of integrity and functionality of the BBB in brains of LOAD patients. A high-throughput multiplex qRT-PCR approach showed transcriptional changes of BBB markers in LOAD BCECs in a cell type-dependent manner. Dysregulation of genes associated with cell-cell contact (including TJs, AJs, intermediate filaments), transport mechanisms across the BBB (including ABC transporters and SLCs), metabolism (including lipoproteins and receptors), and mucins was found in the LOAD model when compared to a matched healthy control. Based on the fact that transcript abundance does not correlate with protein levels in the cell, future studies are required to verify protein expression, localization and function in more detail.

### Electronic supplementary material

Below is the link to the electronic supplementary material.

Supplementary Material 1

Supplementary Material 2

## Data Availability

The authors confirm that the data supporting the findings of this study are available within the article and its supplementary materials. Raw data supporting the findings of this study are available from the corresponding author upon request.

## Abbreviations

Aβ: β-amyloid
ABC: ATP-binding cassette
AC: astrocyte
AD: Alzheimer disease
AJ: adherens junction
BBB: blood-brain barrier
BCEC: brain capillary endothelial-like cell
CON: healthy elderly control
CNS: central nervous system
EC: endothelial cell
hiPSC: human induced pluripotent stem cell
LOAD: late-onset Alzheimer disease
Log_2_FC: logarithm of fold change
MGC: microglia-like cell
NaF: sodium fluorescein
NEM: neural expansion medium
NGD: neuroglial differentiation (medium)
NIM: neural induction medium
NSC: neural stem cell
NVU: neurovascular unit
PC: pericyte
PC_NaF_: permeability coefficient for sodium fluorescein
SD: standard deviation
SEM: standard error of the mean
SLC: solute carrier
STR: short tandem repeat
TEER: transendothelial electrical resistance
TJ: tight junction
tv: transcript variant
