# Supplementary material for: Human isogenic cells of the neurovascular unit exert transcriptomic cell type-specific effects on a blood-brain barrier in vitro model of late-onset Alzheimer disease
Source: Fluids Barriers CNS. 2023 Oct 31;20:78. doi: 10.1186/s12987-023-00471-y (PMC10617216; doi:10.1186/s12987-023-00471-y)
Supplement: Supplementary file 1 — Supplementary Material 1 [file 12987_2023_471_MOESM1_ESM.docx]

**Additional file**

**Title**

**Human isogenic cells of the neurovascular unit exert transcriptomic cell type-specific effects on a blood-brain barrier in vitro model of late-onset Alzheimer disease**

Undine Haferkamp, Carla Hartmann, Chaudry Luqman Abid, Andreas Brachner, Alevtina Höchner, Anna Gerhartl, Bernadette Harwardt, Selin Leckzik, Jennifer Leu, Marco Metzger, Marina Nastainczyk-Wulf, Winfried Neuhaus, Sabrina Oerter, Ole Pless, Dan Rujescu, Matthias Jung, Antje Appelt-Menzel

**Inventory of additional file 1**

Figure S1

Figure S2

Figure S3

Table S1

Table S2

Table S3

# Figure S1


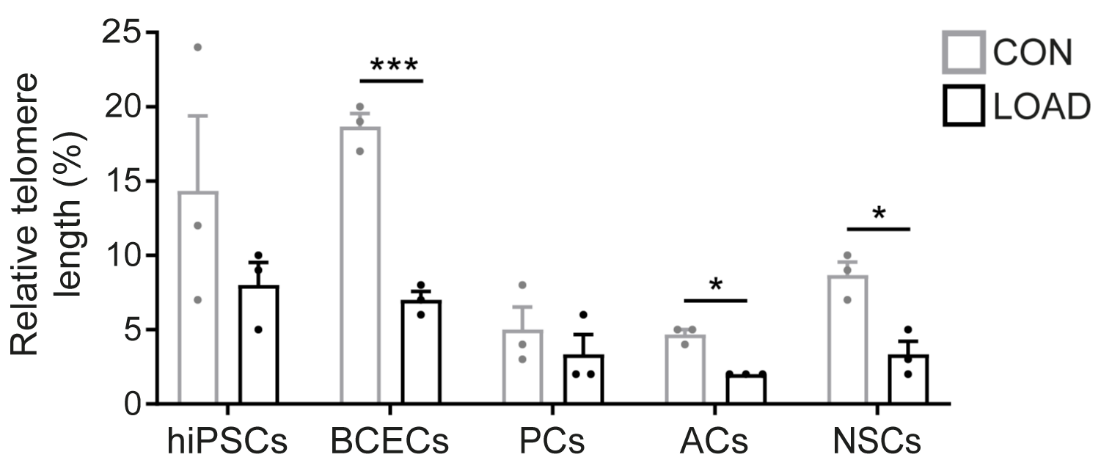


**Telomere length analysis in human iPSC-derived brain cell types of the NVU.** Human induced pluripotent stem cells (hiPSCs) were derived from a late-onset Alzheimer disease patient (LOAD NVU model) and a healthy elderly control subject (CON NVU model). A monochromatic multiplex qPCR was performed to measure the telomere lentgh/single copy gene ratio using albumin (ALB) as a reference. The relative telomere length is shown for hiPSCs, brain capillary endothelial-like cells (BCECs), pericytes (PCs), astrocytes (ACs), and neural stem cells (NSCs) of the CON NVU model and LOAD NVU model. Mean + SEM of n = 3 independent experiments. Unpaired Welch’s t-test, ***p = 0.008 for BCECs, *p = 0.0153 for ACs, *p = 0.0129 for NSCs.

# Figure S2


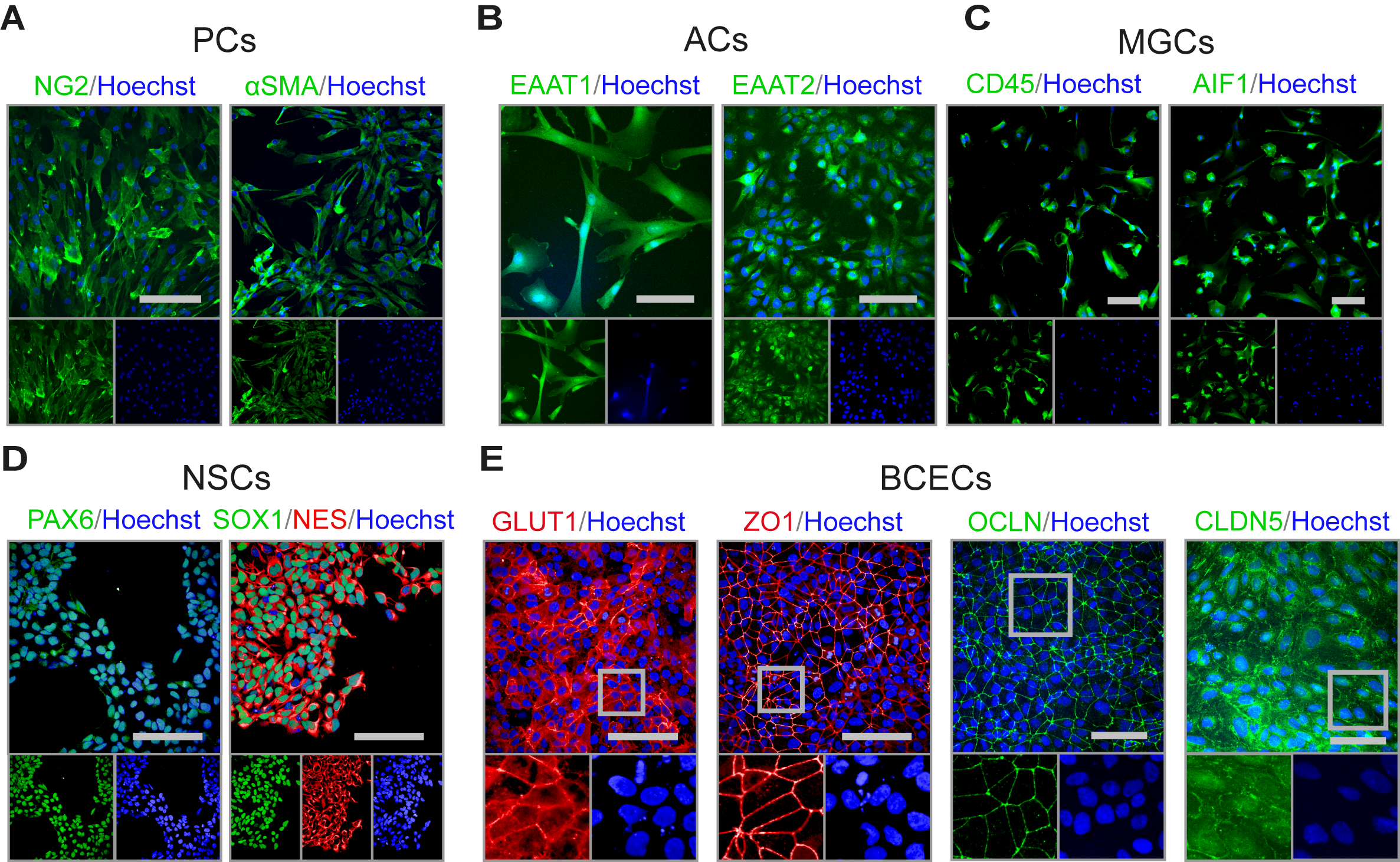


**Expression and localization of marker proteins in the CON hiPSC-derived brain cell types of the NVU.** HiPSCs are derived from a healthy elderly control subject (CON NVU model). Representative immunofluorescence images confirmed the presence of (**A**) NG2 and αSMA in PCs, scale bar 200 µm, (**B**) EAAT1 and EAAT2 in ACs, scale bar 100 µm, (**C**) CD45 and AIF1 in MGCs, scale bar 100 µm, (**D**) PAX6, SOX1, and NES in NSCs, scale bar 100 µm, and (**E**) GLUT1/SLC2A1, ZO1, OCLN, and CLDN5 in BCECs, scale bar 100 µm.

**Figure S3**


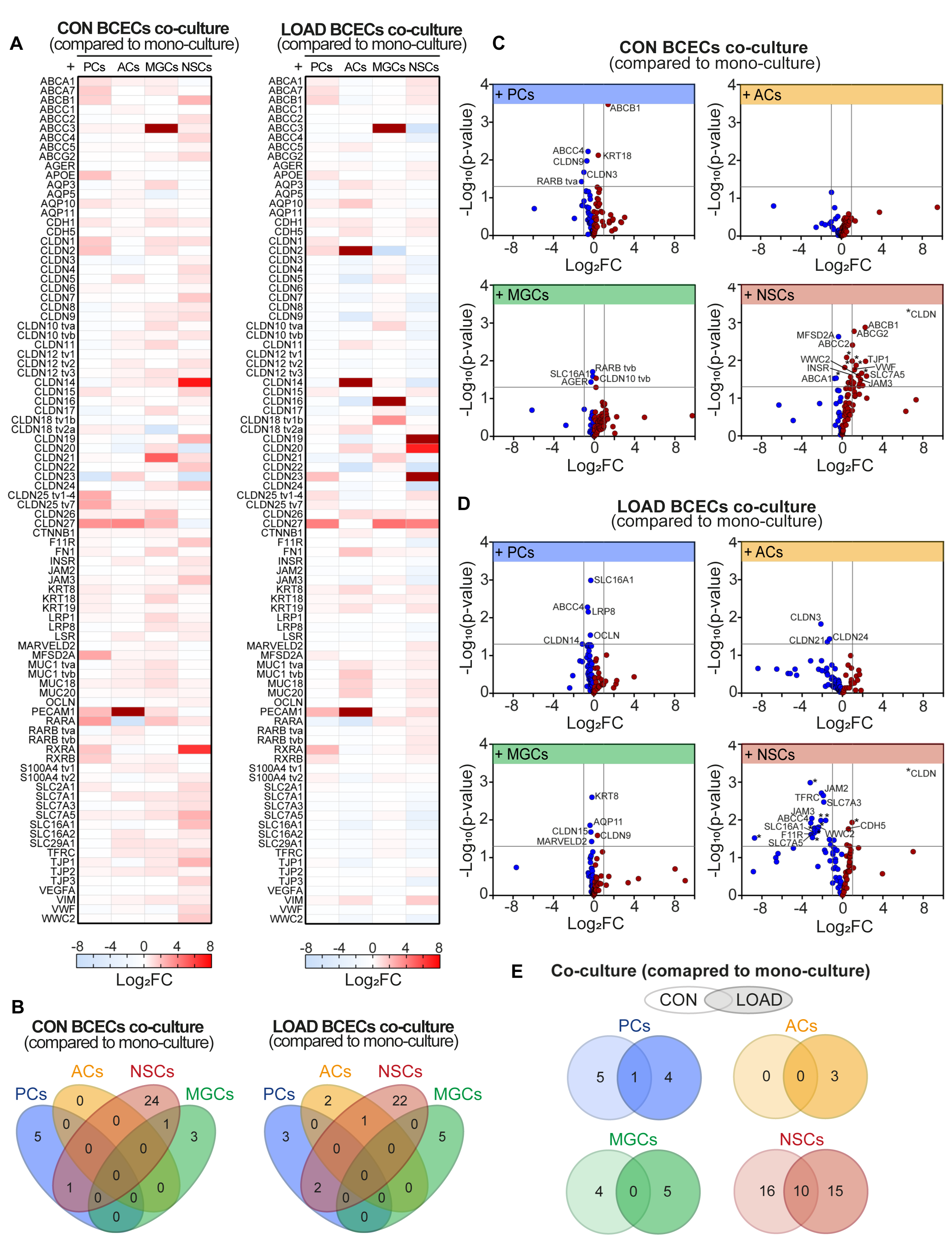


**Transcriptional alterations in LOAD- or CON-BCECs in mono- and co-culture with different NVU cell types.** Comparison of mono- and co-cultures in the CON and LOAD NVU models by high-throughput multiplex qRT-PCR analysis of BBB markers expressed in BCECs (n = 3‑5 independent differentiations). (**A**) HiPSC‑derived CON and LOAD NVU models are displayed separately. The values of the logarithm of fold change (Log_2_FC) for each transcript is plotted on a heat map indicating upregulated (*red*) and downregulated (*blue*) transcripts. (**B**) Venn diagram showing the number of significantly regulated transcripts (p ≤ 0.05) shared between co-cultures with different brain cell types. CON and LOAD NVU models are displayed separately. (**C**) Volcano blot displaying Log_2_FC and p-values for each transcript in the CON NVU model (comparison of CON mono‑ and co‑cultures). Significantly up- (*red*) and downregulated (*blue*) transcripts are shown. (**D**) Volcano blot displaying Log_2_FC and p-values for each transcript in the LOAD NVU model. (**E**) Venn diagram showing the number of significantly regulated transcripts shared between CON and LOAD co-cultures.

# Table S1

Gene target list of high-throughput qPCR. Claudin nomenclature according to Mineta et al. [107].

| **Symbol** | **Target** | **NCBI RefSeq** |
| --- | --- | --- |
| ABCA1 | ATP binding cassette subfamily A member 1 | [NM_005502.4](https://www.ncbi.nlm.nih.gov/nuccore/NM_005502.4) |
| ABCA7 | ATP binding cassette subfamily A member 7 | NM_019112.4 |
| ABCB1 | ATP binding cassette subfamily B member 1, ATP-dependent translocase ABCB1 | NM_000927.4 |
| ABCC1 | ATP binding cassette subfamily C member 1, Multidrug resistance-associated protein 1 | NM_004996.3 |
| ABCC2 | ATP binding cassette subfamily C member 2 | NM_000392.4 |
| ABCC3 | ATP binding cassette subfamily C member 3 | NM_003786.3 |
| ABCC4 | ATP binding cassette subfamily C member 4 | NM_005845.4 |
| ABCC5 | ATP binding cassette subfamily C member 5 | NM_005688.3 |
| ABCG2 | ATP binding cassette subfamily G member 2 | NM_004827.2 |
| ACTB | Actin beta (housekeeping gene) | NM_001101.4 |
| AGER | Advanced glycosylation end product-specific receptor | NM_001136.5,  NM_001206929.2,  NM_001206934.2,  NM_001206936.2,  NM_001206940.2,  NM_001206954.2,  NM_001206966.2 |
| APOE | Apolipoprotein E | NM_000041.4,  NM_001302688.2,  NM_001302689.2,  NM_001302690.1,  NM_001302691.2 |
| AQP3 | Aquaporin 3 | NM_004925.4 |
| AQP5 | Aquaporin 5 | NM_001651.3 |
| AQP10 | Aquaporin 10 | NM_080429.2 |
| AQP11 | Aquaporin 11 | NM_173039.2 |
| B2M | Beta-2-microglobulin (housekeeping gene) | NM_004048.2 |
| CDH1 | Cadherin 1 | NM_001317186.1, NM_001317185.1, NM_001317184., NM_004360.4 |
| CDH5 | Cadherin 5 | NM_001795.4 |
| CLDN1 | Claudin 1 | NM_001307.5 |
| CLDN2 | Claudin 2 | NM_001171095.1 |
| CLDN3 | Claudin 3 | NM_001306.3 |
| CLDN4 | Claudin 4 | NM_001305.4 |
| CLDN5 | Claudin 5 | NM_001130861.1, NM_003277.3 |
| CLDN6 | Claudin 6 | NM_021195.4 |
| CLDN7 | Claudin 7 | NM_001307.5 |
| CLDN8 | Claudin 8 | NM_199328.2 |
| CLDN9 | Claudin 9 | NM_020982.3 |
| CLDN10 tva | Claudin 10 | NM_182848.3 |
| CLDN10 tvb | Claudin 10 | NM_006984.4 |
| CLDN11 | Claudin 11 | NM_005602.5 |
| CLDN12 tv1 | Claudin 12 | NM_001185072.2 |
| CLDN12 tv2 | Claudin 12 | NM_001185073.2 |
| CLDN12 tv3 | Claudin 12 | NM_012129.4 |
| CLDN14 | Claudin 14 | NM_012130.3, NM_001146078.2, NM_001146079.1, NM_001146077.1, NM_144492.2 |
| CLDN15 | Claudin 15 | NM_014343.2,  NM_001185080.1 |
| CLDN16 | Claudin 16 | NM_006580.3 |
| CLDN17 | Claudin 17 | NM_012131.2 |
| CLDN18 tv1b | Claudin 18 | NM_016369.3 |
| CLDN18 tv2a | Claudin 18 | NM_001002026.2 |
| CLDN19 | Claudin 19 | NM_148960.2 |
| CLDN20 | Claudin 20 | NM_001001346.3 |
| CLDN21 | CLDN25, Claudin 21 according to Mineta et al. [107] | NM_001101389.1 |
| CLDN22 | Claudin 22 | NM_001111319.1 |
| CLDN23 | Claudin 23 | NM_194284.2 |
| CLDN24 | Claudin 24 | NM_001185149.1 |
| CLDN25 tv1-4, tv6 | CLDN1, Claudin 25 according to Mineta et al. [107] | NM_001040181.2  NM_019895.3  NM_001040183.2  NM_001040182.2  NM_001040199.2 |
| CLDN25 tv7 | CLDN1, Claudin 25 according to Mineta et al. [107] | NM_001040200.2 |
| CLDN26 | Claudin 26 according to Mineta et al. [107] | NM_001146336.1 |
| CLDN27 | Claudin 27 according to Mineta et al. [107] | NM_001204210.1, NM_001204211.1, NM_001204212.1 |
| CTNNB1 | Catenin beta-1 | NM_001098209.1, NM_001904.3, NM_001098210.1 |
| F11R | F11 receptor, Junctional adhesion molecule A | NM_016946.4 |
| FN1 | Fibronectin | NM_212474.2, NM_212476.2, NM_212478.2, NM_002026.3, NM_212482.2, NM_001306132.1, NM_001306131 |
| GAPDH | Glyceraldehyde-3-phosphate dehydrogenase (housekeeping gene) | NM_002046 |
| INSR | Insulin receptor | NM_001079817.2, NM_000208.3 |
| JAM2 | Junctional adhesion molecule 2 | NM_021219.3 |
| JAM3 | Junctional adhesion molecule 3 | NM_032801.4 |
| KRT8 | Keratin 8 | NM_001256293.1, NM_001256282.1 |
| KRT18 | Keratin 18 | NM_199187.1,  NM_000224.2 |
| KRT19 | Keratin 19 | NM_002276.4 |
| LRP1 | LDL receptor related protein 1, Prolow-density lipoprotein receptor-related protein 1 | NM_002332.2 |
| LRP8 | LDL receptor related protein 8, Low-density lipoprotein receptor-related protein 8 | NM_004631.4, NM_001018054.2, NM_033300.3, NM_017522.4 |
| LSR | Lipolysis-stimulated lipoprotein receptor | NM_001260490.2,  NM_015925.7,  NM_205834.4,  NM_205835.4 |
| MARVELD2 | MARVEL domain containing 2, MARVEL domain-containing protein 2 | NM_001244734.1, NM_001038603.2 |
| MFSD2A | Major facilitator superfamily domain containing 2A, Sodium-dependent lysophosphatidylcholine symporter 1 | NM_001349821.1, NM_001349823.1, NM_001349822.1, NM_032793.4, NM_001136493.2, NM_001287809.1, NM_0012878 |
| MUC1 tva | Mucin 1 | NM_001204294.1, NM_001204293.1, NM_001204285.1, NM_001018017.2, NM_001044390.2, NM_00104439 |
| MUC1 tvb | Mucin 1 | NM_001204296.1, NM_001204297.1, NM_001204295.1, NM_001204292.1, NM_001204291.1, NM_001204289. |
| MUC18 | Mucin 18 | NM_006500.2 |
| MUC20 | Mucin 20 | NM_152673.3, NM_001291833.1, NM_020790.1, NM_001282506.1 |
| OCLN | Occludin | NM_001205255.1, NM_001205254.1, NM_002538.3 |
| PECAM1 | Platelet endothelial cell adhesion molecule 1 | NM_000442.5 |
| PPIA | Peptidylprolyl isomerase A (housekeeping gene) | NM_021130.4 |
| RARA | Retinoic acid receptor alpha | NM_000964.4,  NM_001024809.4,  NM_001145301.3,  NM_001145302.3 |
| RARB tva | Retinoic acid receptor beta | NM_000965.4,  NM_016152.3,  NM_001290216.2,  NM_001290217.1,  NM_001290300 |
| RARB tvb | Retinoic acid receptor beta | NM_000965.4,  NM_016152.3,  NM_001290277.1 |
| RXRA | Retinoic acid receptor RXR-alpha | NM_002957.6 |
| RXRB | Retinoic acid receptor RXR-beta | NM_021976.5 |
| S100A4 tv1 | S100 calcium binding protein A4 | NM_002961.2 |
| S100A4 tv2 | S100 calcium binding protein A4 | NM_019554.2 |
| SLC2A1 | Solute carrier family 2 member 1, Solute carrier family 2, facilitated glucose transporter member 1 | NM_006516.2 |
| SLC7A1 | Solute carrier family 7 member 1, High affinity cationic amino acid transporter 1 | NM_003045.4 |
| SLC7A3 | Solute carrier family 7 member 3, Cationic amino acid transporter 3 | NM_032803.5 |
| SLC7A5 | Solute carrier family 7 member 5, Large neutral amino acids transporter small subunit 1 | NM_003486.6 |
| SLC16A1 | Solute carrier family 16 member 1, Monocarboxylate transporter 1 | NM_003051.3, NM_001166496.1 |
| SLC16A2 | Solute carrier family 16 member 2, Monocarboxylate transporter 8 | NM_006517.4 |
| SLC29A1 | Solute carrier family 29 member 1, Equilibrative nucleoside transporter 1 | NM_001078175.2, NM_001078177.1, NM_001304463.1, NM_001304462.1 |
| TFRC | Transferrin receptor protein 1 | NM_001313966.1, NM_001313965.1, NM_003234.3, NM_001128148.2 |
| TJP1 | Tight junction protein 1 | NM_003257.4 |
| TJP2 | Tight junction protein 2 | NM_001170414.2, NM_201629.3, NM_001170416.1, NM_001170415.1, NM_004817.3 |
| TJP3 | Tight junction protein 3 | NM_001267561.1 |
| VEGFA | Vascular endothelial growth factor A | NM_001204384.1, NM_001171622.1, NM_001033756.2, NM_001025370.2, NM_001025369.2, NM_001025368 |
| VIM | Vimentin | NM_003380.4 |
| VWF | von Willebrand factor | NM_000552 |
| WWC2 | WW-and-C2-domain-containing family of proteins | NM_024949.5 |

**Table S2**

Results STR analysis

| **Cell type** | CON (MLUi009-A) | | | | | LOAD (MLUi007-J) | | | | |
| --- | --- | --- | --- | --- | --- | --- | --- | --- | --- | --- |
| **Locus** | hiPSCs | ACs | BCECs | NSCs | PCs | hiPSCs | ACs | BCECs | NSCs | PCs |
| AMEL | X | X | X | X | X | X | X | X | X | X |
| D3S1358 | 14, 16 | 14, 16 | 14, 16 | 14, 16 | 14, 16 | 14, 15 | 14, 15 | 14, 15 | 14, 15 | 14, 15 |
| TH01 | 8, 9 | 8, 9 | 8, 9 | 8, 9 | 8, 9 | 7, 9 | 7, 9 | 7, 9 | 7, 9 | 7, 9 |
| D21S11 | 30, 31.2 | 30, 31.2 | 30, 31.2 | 30, 31.2 | 30, 31.2 | 28, 29.2 | 28, 29.2 | 28, 29.2 | 28, 29.2 | 28, 29.2 |
| D18S51 | 12, 18 | 12, 18 | 12, 18 | 12, 18 | 12, 18 | 16, 17 | 16, 17 | 16, 17 | 16, 17 | 16, 17 |
| D10S1248 | 13, 14 | 13, 14 | 13, 14 | 13, 14 | 13, 14 | 14, 18 | 14, 18 | 14, 18 | 14, 18 | 14, 18 |
| D1S1656 | 14, 17.3 | 14, 17.3 | 14, 17.3 | 14, 17.3 | 14, 17.3 | 15.3, 17.3 | 15.3, 17.3 | 15.3, 17.3 | 15.3, 17.3 | 15.3, 17.3 |
| D2S1338 | 16, 17 | 16, 17 | 16, 17 | 16, 17 | 16, 17 | 16, 20 | 16, 20 | 16, 20 | 16, 20 | 16, 20 |
| D16S539 | 9, 11 | 9, 11 | 9, 11 | 9, 11 | 9, 11 | 8, 9 | 8, 9 | 8, 9 | 8, 9 | 8, 9 |
| D22S1045 | 15 | 15 | 15 | 15 | 15 | 11, 15 | 11, 15 | 11, 15 | 11, 15 | 11, 15 |
| vWA | 15, 19 | 15, 19 | 15, 19 | 15, 19 | 15, 19 | 15, 17 | 15, 17 | 15, 17 | 15, 17 | 15, 17 |
| D8S1179 | 13, 15 | 13, 15 | 13, 15 | 13, 15 | 13, 15 | 13 | 13 | 13 | 13 | 13 |
| FGA | 20, 24 | 20, 24 | 20, 24 | 20, 24 | 20, 24 | 19, 23 | 19, 23 | 19, 23 | 19, 23 | 19, 23 |
| D2S441 | 10, 14 | 10, 14 | 10, 14 | 10, 14 | 10, 14 | 10.1, 15 | 10.1, 15 | 10.1, 15 | 10.1, 15 | 10.1, 15 |
| D12S391 | 18, 24 | 18, 24 | 18, 24 | 18, 24 | 18, 24 | 15, 25 | 15, 25 | 15, 25 | 15, 25 | 15, 25 |
| D19S433 | 14, 15.2 | 14, 15.2 | 14, 15.2 | 14, 15.2 | 14, 15.2 | 13, 15.2 | 13, 15.2 | 13, 15.2 | 13, 15.2 | 13, 15.2 |
| SE33 | 19, 32.2 | 19, 32.2 | 19, 32.2 | 19, 32.2 | 19, 32.2 | 19, 29.2 | 19, 29.2 | 19, 29.2 | 19, 29.2 | 19, 29.2 |
|  | Matching female DNA profile | | | | | Matching female DNA profile | | | | |

**Table S3**

TEER measurements and sodium fluorescein permeability coefficient (PC_NaF_) measurements on LOAD and CON BCECs in co-culture. All values are provided as mean ± SEM, n = 4-6.

| **TEER (Ω*cm2)** | | |
| --- | --- | --- |
| **CON** | **Mono-culture** | **Co-culture** |
| PCs | 1178 $\pm$ 477 | 1896 $\pm$ 339 |
| ACs | 2040 $\pm$ 223 | 2214 $\pm$ 217 |
| MGCs | 1943 $\pm$ 134 | 1834 $\pm$ 103 |
| NSCs | 2299 $\pm$ 123 | 2674 $\pm$ 670 |
| **LOAD** | **Mono-culture** | **Co-culture** |
| PCs | 1400 $\pm$ 189 | 1957 $\pm$ 284 |
| ACs | 1000 $\pm$ 148 | 924 $\pm$ 168 |
| MGCs | 1091 $\pm$ 208 | 990 $\pm$ 137 |
| NSCs | 2108 $\pm$ 163 | 2310 $\pm$ 111 |
| **PC_NaF_ (**$\boldsymbol{\mu}$**m / min)** | | |
| **CON** | **Mono-culture** | **Co-culture** |
| PCs | 0.870 $\pm$ 0.235 | 0.438 $\pm$ 0.091 |
| ACs | 0.596 $\pm$ 0.103 | 0.564 $\pm$ 0.066 |
| MGCs | 1.080 $\pm$ 0.088 | 0.840 $\pm$ 0.111 |
| NSCs | 0.472 $\pm$ 0.197 | 0.432 $\pm$ 0.142 |
| **LOAD** | **Mono-culture** | **Co-culture** |
| PCs | 0.890 $\pm$ 0.258 | 0.608 $\pm$ 0.050 |
| ACs | 0.726 $\pm$ 0.135 | 0.728 $\pm$ 0.181 |
| MGCs | 0.955 $\pm$ 0.238 | 0.853 $\pm$ 0.206 |
| NSCs | 0.238 $\pm$ 0.086 | 0.182 $\pm$ 0.025 |

TEER: transendothelial electrical resistance; PC: permeability coefficient
