# Supplementary material for: Human isogenic cells of the neurovascular unit exert transcriptomic cell type-specific effects on a blood-brain barrier in vitro model of late-onset Alzheimer disease
Source: Fluids Barriers CNS. 2023 Oct 31;20:78. doi: 10.1186/s12987-023-00471-y (PMC10617216; doi:10.1186/s12987-023-00471-y)
Supplement: Supplementary file 2 — Supplementary Material 2 [file 12987_2023_471_MOESM2_ESM.docx]

**Additional file**

**Title**

**Human isogenic cells of the neurovascular unit exert transcriptomic cell type-specific effects on a blood-brain barrier *in vitro* model of late-onset Alzheimer disease**

Undine Haferkamp, Carla Hartmann, Chaudhry Luqman Abid, Andreas Brachner, Alevtina Höchner, Anna Gerhartl, Bernadette Harwardt, Selin Leckzik, Jennifer Leu, Marco Metzger, Marina Nastainczyk-Wulf, Winfried Neuhaus, Sabrina Oerter, Ole Pless, Dan Rujescu, Matthias Jung, Antje Appelt-Menzel

**Inventory of additional file 1**

Figure S1

Figure S2

Table S1

Table S2

Table S3

# Figure S1


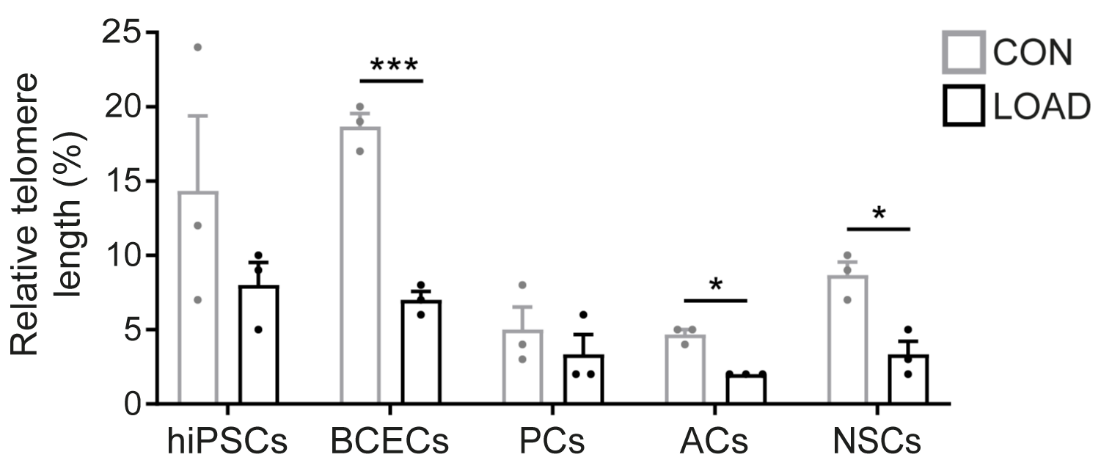


**Telomere length analysis in hiPSC-derived brain cell types of the NVU.** Human induced pluripotent stem cells (hiPSCs) were derived from a late-onset Alzheimer disease patient (LOAD NVU model) and a healthy elderly control subject (CON NVU model). A monochromatic multiplex qPCR was performed to measure the telomere lentgh/single copy gene ratio using albumin (ALB) as a reference. The relative telomere length is shown for hiPSCs, brain capillary endothelial-like cells (BCECs), pericytes (PCs), astrocytes (ACs), and neural stem cells (NSCs) of the CON NVU model and LOAD NVU model. Mean + SEM of n = 3 independent experiments. Unpaired Welch’s t-test, ***p = 0.008 for BCECs, *p = 0.0153 for ACs, *p = 0.0129 for NSCs.

# Figure S2


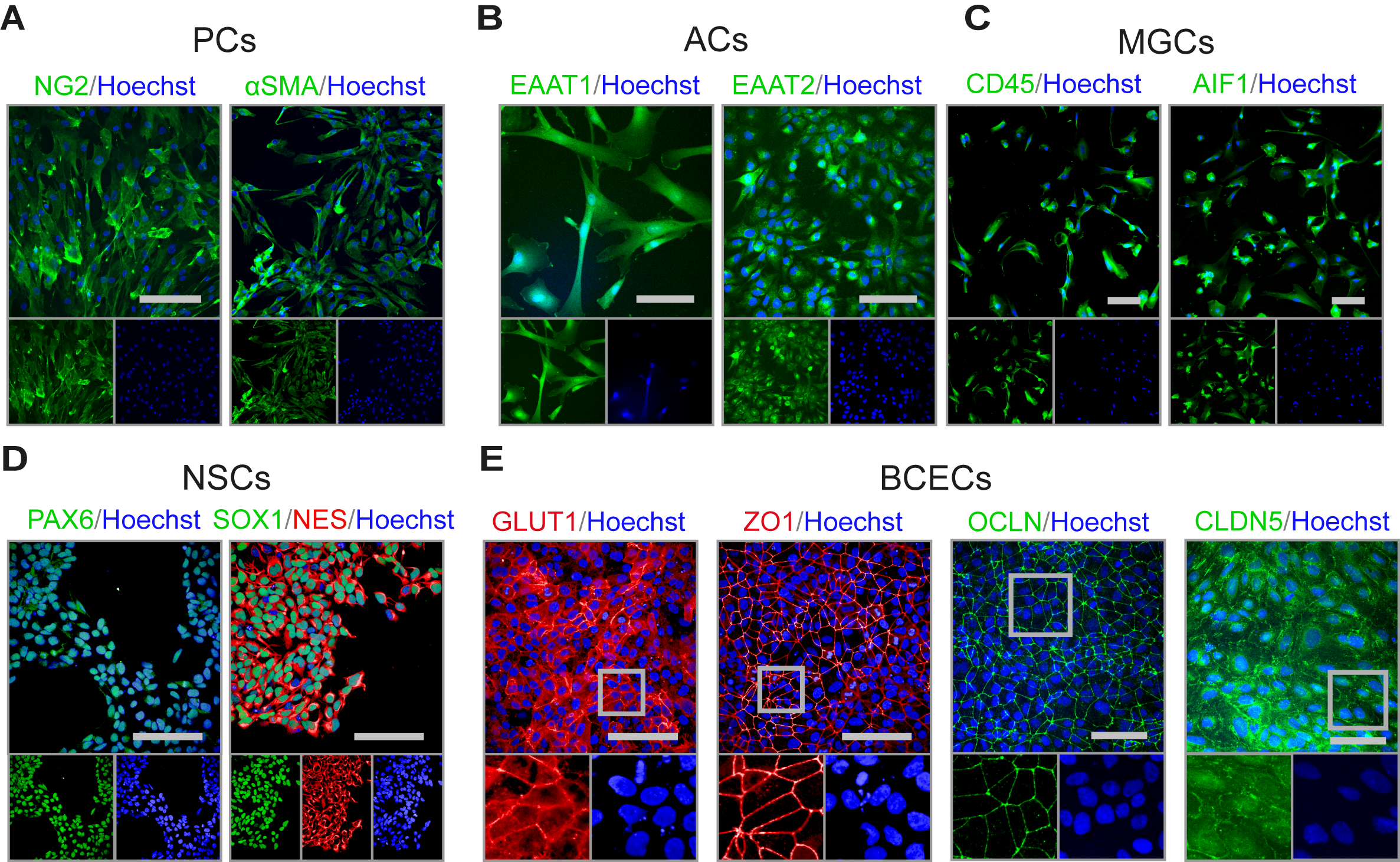


**Expression and localization of marker proteins in the CON hiPSC-derived brain cell types of the NVU.** HiPSCs are derived from a healthy elderly control subject (CON NVU model). Representative immunofluorescence images confirmed the presence of (**A**) NG2 and αSMA in PCs, scale bar 200 µm, (**B**) EAAT1 and EAAT2 in ACs, scale bar 100 µm, (**C**) CD45 and AIF1 in MGCs, scale bar 100 µm, (**D**) PAX6, SOX1, and NES in NSCs, scale bar 100 µm, and (**E**) GLUT1/SLC2A1, ZO1, OCLN, and CLDN5 in BCECs, scale bar 100 µm.

# Table S1

Gene target list of high-throughput qPCR. Claudin nomenclature according to Mineta et al. 2011^1^.

| **Symbol** | **Target** | **NCBI RefSeq** |
| --- | --- | --- |
| ABCA1 | ATP binding cassette subfamily A member 1 | [NM_005502.4](https://www.ncbi.nlm.nih.gov/nuccore/NM_005502.4) |
| ABCA7 | ATP binding cassette subfamily A member 7 | NM_019112.4 |
| ABCB1 | ATP binding cassette subfamily B member 1, ATP-dependent translocase ABCB1 | NM_000927.4 |
| ABCC1 | ATP binding cassette subfamily C member 1, Multidrug resistance-associated protein 1 | NM_004996.3 |
| ABCC2 | ATP binding cassette subfamily C member 2 | NM_000392.4 |
| ABCC3 | ATP binding cassette subfamily C member 3 | NM_003786.3 |
| ABCC4 | ATP binding cassette subfamily C member 4 | NM_005845.4 |
| ABCC5 | ATP binding cassette subfamily C member 5 | NM_005688.3 |
| ABCG2 | ATP binding cassette subfamily G member 2 | NM_004827.2 |
| ACTB | Actin beta (housekeeping gene) | NM_001101.4 |
| AGER | Advanced glycosylation end product-specific receptor | NM_001136.5,  NM_001206929.2,  NM_001206934.2,  NM_001206936.2,  NM_001206940.2,  NM_001206954.2,  NM_001206966.2 |
| APOE | Apolipoprotein E | NM_000041.4,  NM_001302688.2,  NM_001302689.2,  NM_001302690.1,  NM_001302691.2 |
| AQP3 | Aquaporin 3 | NM_004925.4 |
| AQP5 | Aquaporin 5 | NM_001651.3 |
| AQP10 | Aquaporin 10 | NM_080429.2 |
| AQP11 | Aquaporin 11 | NM_173039.2 |
| B2M | Beta-2-microglobulin (housekeeping gene) | NM_004048.2 |
| CDH1 | Cadherin 1 | NM_001317186.1, NM_001317185.1, NM_001317184., NM_004360.4 |
| CDH5 | Cadherin 5 | NM_001795.4 |
| CLDN1 | Claudin 1 | NM_001307.5 |
| CLDN2 | Claudin 2 | NM_001171095.1 |
| CLDN3 | Claudin 3 | NM_001306.3 |
| CLDN4 | Claudin 4 | NM_001305.4 |
| CLDN5 | Claudin 5 | NM_001130861.1, NM_003277.3 |
| CLDN6 | Claudin 6 | NM_021195.4 |
| CLDN7 | Claudin 7 | NM_001307.5 |
| CLDN8 | Claudin 8 | NM_199328.2 |
| CLDN9 | Claudin 9 | NM_020982.3 |
| CLDN10 tva | Claudin 10 | NM_182848.3 |
| CLDN10 tvb | Claudin 10 | NM_006984.4 |
| CLDN11 | Claudin 11 | NM_005602.5 |
| CLDN12 tv1 | Claudin 12 | NM_001185072.2 |
| CLDN12 tv2 | Claudin 12 | NM_001185073.2 |
| CLDN12 tv3 | Claudin 12 | NM_012129.4 |
| CLDN14 | Claudin 14 | NM_012130.3, NM_001146078.2, NM_001146079.1, NM_001146077.1, NM_144492.2 |
| CLDN15 | Claudin 15 | NM_014343.2,  NM_001185080.1 |
| CLDN16 | Claudin 16 | NM_006580.3 |
| CLDN17 | Claudin 17 | NM_012131.2 |
| CLDN18 tv1b | Claudin 18 | NM_016369.3 |
| CLDN18 tv2a | Claudin 18 | NM_001002026.2 |
| CLDN19 | Claudin 19 | NM_148960.2 |
| CLDN20 | Claudin 20 | NM_001001346.3 |
| CLDN21 | CLDN25, Claudin 21 according to Mineta et al. [107] | NM_001101389.1 |
| CLDN22 | Claudin 22 | NM_001111319.1 |
| CLDN23 | Claudin 23 | NM_194284.2 |
| CLDN24 | Claudin 24 | NM_001185149.1 |
| CLDN25 tv1-4, tv6 | CLDN1, Claudin 25 according to Mineta et al. [107] | NM_001040181.2  NM_019895.3  NM_001040183.2  NM_001040182.2  NM_001040199.2 |
| CLDN25 tv7 | CLDN1, Claudin 25 according to Mineta et al. [107] | NM_001040200.2 |
| CLDN26 | Claudin 26 according to Mineta et al. [107] | NM_001146336.1 |
| CLDN27 | Claudin 27 according to Mineta et al. [107] | NM_001204210.1, NM_001204211.1, NM_001204212.1 |
| CTNNB1 | Catenin beta-1 | NM_001098209.1, NM_001904.3, NM_001098210.1 |
| F11R | F11 receptor, Junctional adhesion molecule A | NM_016946.4 |
| FN1 | Fibronectin | NM_212474.2, NM_212476.2, NM_212478.2, NM_002026.3, NM_212482.2, NM_001306132.1, NM_001306131 |
| GAPDH | Glyceraldehyde-3-phosphate dehydrogenase (housekeeping gene) | NM_002046 |
| INSR | Insulin receptor | NM_001079817.2, NM_000208.3 |
| JAM2 | Junctional adhesion molecule 2 | NM_021219.3 |
| JAM3 | Junctional adhesion molecule 3 | NM_032801.4 |
| KRT8 | Keratin 8 | NM_001256293.1, NM_001256282.1 |
| KRT18 | Keratin 18 | NM_199187.1,  NM_000224.2 |
| KRT19 | Keratin 19 | NM_002276.4 |
| LRP1 | LDL receptor related protein 1, Prolow-density lipoprotein receptor-related protein 1 | NM_002332.2 |
| LRP8 | LDL receptor related protein 8, Low-density lipoprotein receptor-related protein 8 | NM_004631.4, NM_001018054.2, NM_033300.3, NM_017522.4 |
| LSR | Lipolysis-stimulated lipoprotein receptor | NM_001260490.2,  NM_015925.7,  NM_205834.4,  NM_205835.4 |
| MARVELD2 | MARVEL domain containing 2, MARVEL domain-containing protein 2 | NM_001244734.1, NM_001038603.2 |
| MFSD2A | Major facilitator superfamily domain containing 2A, Sodium-dependent lysophosphatidylcholine symporter 1 | NM_001349821.1, NM_001349823.1, NM_001349822.1, NM_032793.4, NM_001136493.2, NM_001287809.1, NM_0012878 |
| MUC1 tva | Mucin 1 | NM_001204294.1, NM_001204293.1, NM_001204285.1, NM_001018017.2, NM_001044390.2, NM_00104439 |
| MUC1 tvb | Mucin 1 | NM_001204296.1, NM_001204297.1, NM_001204295.1, NM_001204292.1, NM_001204291.1, NM_001204289. |
| MUC18 | Mucin 18 | NM_006500.2 |
| MUC20 | Mucin 20 | NM_152673.3, NM_001291833.1, NM_020790.1, NM_001282506.1 |
| OCLN | Occludin | NM_001205255.1, NM_001205254.1, NM_002538.3 |
| PECAM1 | Platelet endothelial cell adhesion molecule 1 | NM_000442.5 |
| PPIA | Peptidylprolyl isomerase A (housekeeping gene) | NM_021130.4 |
| RARA | Retinoic acid receptor alpha | NM_000964.4,  NM_001024809.4,  NM_001145301.3,  NM_001145302.3 |
| RARB tva | Retinoic acid receptor beta | NM_000965.4,  NM_016152.3,  NM_001290216.2,  NM_001290217.1,  NM_001290300 |
| RARB tvb | Retinoic acid receptor beta | NM_000965.4,  NM_016152.3,  NM_001290277.1 |
| RXRA | Retinoic acid receptor RXR-alpha | NM_002957.6 |
| RXRB | Retinoic acid receptor RXR-beta | NM_021976.5 |
| S100A4 tv1 | S100 calcium binding protein A4 | NM_002961.2 |
| S100A4 tv2 | S100 calcium binding protein A4 | NM_019554.2 |
| SLC2A1 | Solute carrier family 2 member 1, Solute carrier family 2, facilitated glucose transporter member 1 | NM_006516.2 |
| SLC7A1 | Solute carrier family 7 member 1, High affinity cationic amino acid transporter 1 | NM_003045.4 |
| SLC7A3 | Solute carrier family 7 member 3, Cationic amino acid transporter 3 | NM_032803.5 |
| SLC7A5 | Solute carrier family 7 member 5, Large neutral amino acids transporter small subunit 1 | NM_003486.6 |
| SLC16A1 | Solute carrier family 16 member 1, Monocarboxylate transporter 1 | NM_003051.3, NM_001166496.1 |
| SLC16A2 | Solute carrier family 16 member 2, Monocarboxylate transporter 8 | NM_006517.4 |
| SLC29A1 | Solute carrier family 29 member 1, Equilibrative nucleoside transporter 1 | NM_001078175.2, NM_001078177.1, NM_001304463.1, NM_001304462.1 |
| TFRC | Transferrin receptor protein 1 | NM_001313966.1, NM_001313965.1, NM_003234.3, NM_001128148.2 |
| TJP1 | Tight junction protein 1 | NM_003257.4 |
| TJP2 | Tight junction protein 2 | NM_001170414.2, NM_201629.3, NM_001170416.1, NM_001170415.1, NM_004817.3 |
| TJP3 | Tight junction protein 3 | NM_001267561.1 |
| VEGFA | Vascular endothelial growth factor A | NM_001204384.1, NM_001171622.1, NM_001033756.2, NM_001025370.2, NM_001025369.2, NM_001025368 |
| VIM | Vimentin | NM_003380.4 |
| VWF | von Willebrand factor | NM_000552 |
| WWC2 | WW-and-C2-domain-containing family of proteins | NM_024949.5 |

^1^Mineta K, Yamamoto Y, Yamazaki Y, Tanaka H, Tada Y, Saito K, Tamura A, Igarashi M, Endo T, Takeuchi K, Tsukita S. Predicted expansion of the claudin multigene family. FEBS Lett. 2011 Feb 18;585(4):606-12. doi: 10.1016/j.febslet.2011.01.028. Epub 2011 Jan 26. PMID: 21276448

**Table S2**

Results short tandem repeat (STR) analysis.

| **Cell type** | CON (MLUi009-A) | | | | | LOAD (MLUi007-J) | | | | |
| --- | --- | --- | --- | --- | --- | --- | --- | --- | --- | --- |
| **Locus** | hiPSCs | ACs | BCECs | NSCs | PCs | hiPSCs | ACs | BCECs | NSCs | PCs |
| AMEL | X | X | X | X | X | X | X | X | X | X |
| D3S1358 | 14, 16 | 14, 16 | 14, 16 | 14, 16 | 14, 16 | 14, 15 | 14, 15 | 14, 15 | 14, 15 | 14, 15 |
| TH01 | 8, 9 | 8, 9 | 8, 9 | 8, 9 | 8, 9 | 7, 9 | 7, 9 | 7, 9 | 7, 9 | 7, 9 |
| D21S11 | 30, 31.2 | 30, 31.2 | 30, 31.2 | 30, 31.2 | 30, 31.2 | 28, 29.2 | 28, 29.2 | 28, 29.2 | 28, 29.2 | 28, 29.2 |
| D18S51 | 12, 18 | 12, 18 | 12, 18 | 12, 18 | 12, 18 | 16, 17 | 16, 17 | 16, 17 | 16, 17 | 16, 17 |
| D10S1248 | 13, 14 | 13, 14 | 13, 14 | 13, 14 | 13, 14 | 14, 18 | 14, 18 | 14, 18 | 14, 18 | 14, 18 |
| D1S1656 | 14, 17.3 | 14, 17.3 | 14, 17.3 | 14, 17.3 | 14, 17.3 | 15.3, 17.3 | 15.3, 17.3 | 15.3, 17.3 | 15.3, 17.3 | 15.3, 17.3 |
| D2S1338 | 16, 17 | 16, 17 | 16, 17 | 16, 17 | 16, 17 | 16, 20 | 16, 20 | 16, 20 | 16, 20 | 16, 20 |
| D16S539 | 9, 11 | 9, 11 | 9, 11 | 9, 11 | 9, 11 | 8, 9 | 8, 9 | 8, 9 | 8, 9 | 8, 9 |
| D22S1045 | 15 | 15 | 15 | 15 | 15 | 11, 15 | 11, 15 | 11, 15 | 11, 15 | 11, 15 |
| vWA | 15, 19 | 15, 19 | 15, 19 | 15, 19 | 15, 19 | 15, 17 | 15, 17 | 15, 17 | 15, 17 | 15, 17 |
| D8S1179 | 13, 15 | 13, 15 | 13, 15 | 13, 15 | 13, 15 | 13 | 13 | 13 | 13 | 13 |
| FGA | 20, 24 | 20, 24 | 20, 24 | 20, 24 | 20, 24 | 19, 23 | 19, 23 | 19, 23 | 19, 23 | 19, 23 |
| D2S441 | 10, 14 | 10, 14 | 10, 14 | 10, 14 | 10, 14 | 10.1, 15 | 10.1, 15 | 10.1, 15 | 10.1, 15 | 10.1, 15 |
| D12S391 | 18, 24 | 18, 24 | 18, 24 | 18, 24 | 18, 24 | 15, 25 | 15, 25 | 15, 25 | 15, 25 | 15, 25 |
| D19S433 | 14, 15.2 | 14, 15.2 | 14, 15.2 | 14, 15.2 | 14, 15.2 | 13, 15.2 | 13, 15.2 | 13, 15.2 | 13, 15.2 | 13, 15.2 |
| SE33 | 19, 32.2 | 19, 32.2 | 19, 32.2 | 19, 32.2 | 19, 32.2 | 19, 29.2 | 19, 29.2 | 19, 29.2 | 19, 29.2 | 19, 29.2 |
|  | Matching female DNA profile | | | | | Matching female DNA profile | | | | |

**Table S3**

TEER measurements and sodium fluorescein permeability coefficient (PC_NaF_) measurements on LOAD and CON BCECs in co-culture. All values are provided as mean ± SEM, n = 4-6.

| **TEER (Ω*cm^2^)** | | |
| --- | --- | --- |
| **CON** | **Mono-culture** | **Co-culture** |
| PCs | 1178 $\pm$ 477 | 1896 $\pm$ 339 |
| ACs | 2040 $\pm$ 223 | 2214 $\pm$ 217 |
| MGCs | 1943 $\pm$ 134 | 1834 $\pm$ 103 |
| NSCs | 2299 $\pm$ 123 | 2674 $\pm$ 670 |
| **LOAD** | **Mono-culture** | **Co-culture** |
| PCs | 1400 $\pm$ 189 | 1957 $\pm$ 284 |
| ACs | 1000 $\pm$ 148 | 924 $\pm$ 168 |
| MGCs | 1091 $\pm$ 208 | 990 $\pm$ 137 |
| NSCs | 2108 $\pm$ 163 | 2310 $\pm$ 111 |
| **PC_NaF_ (**$\boldsymbol{\mu}$**m/min)** | | |
| **CON** | **Mono-culture** | **Co-culture** |
| PCs | 0.870 $\pm$ 0.235 | 0.438 $\pm$ 0.091 |
| ACs | 0.596 $\pm$ 0.103 | 0.564 $\pm$ 0.066 |
| MGCs | 1.080 $\pm$ 0.088 | 0.840 $\pm$ 0.111 |
| NSCs | 0.472 $\pm$ 0.197 | 0.432 $\pm$ 0.142 |
| **LOAD** | **Mono-culture** | **Co-culture** |
| PCs | 0.890 $\pm$ 0.258 | 0.608 $\pm$ 0.050 |
| ACs | 0.726 $\pm$ 0.135 | 0.728 $\pm$ 0.181 |
| MGCs | 0.955 $\pm$ 0.238 | 0.853 $\pm$ 0.206 |
| NSCs | 0.238 $\pm$ 0.086 | 0.182 $\pm$ 0.025 |

TEER: transendothelial electrical resistance; PC: permeability coefficient
